# Systematic Analysis of the Impact of R-Methylation on RBPs-RNA Interactions: A Proteomic Approach

**DOI:** 10.3389/fmolb.2021.688973

**Published:** 2021-09-07

**Authors:** Marianna Maniaci, Francesca Ludovica Boffo, Enrico Massignani, Tiziana Bonaldi

**Affiliations:** ^1^ Laboratory of Nuclear Proteomics to Study Gene Expression Regulation in Cancer, European Institute of Oncology (IEO) IRCSS, Department of Experimental Oncology (DEO), Milan, Italy; ^2^ European School of Molecular Medicine (SEMM), Milan, Italy

**Keywords:** proteomics, PTMs, protein-R-methylation, PRMTs, SILAC, OOPS, RBPs, mass spectrometry

## Abstract

RNA binding proteins (RBPs) bind RNAs through specific RNA-binding domains, generating multi-molecular complexes known as ribonucleoproteins (RNPs). Various post-translational modifications (PTMs) have been described to regulate RBP structure, subcellular localization, and interactions with other proteins or RNAs. Recent proteome-wide experiments showed that RBPs are the most representative group within the class of arginine (R)-methylated proteins. Moreover, emerging evidence suggests that this modification plays a role in the regulation of RBP-RNA interactions. Nevertheless, a systematic analysis of how changes in protein-R-methylation can affect globally RBPs-RNA interactions is still missing. We describe here a quantitative proteomics approach to profile global changes of RBP-RNA interactions upon the modulation of type I and II protein arginine methyltransferases (PRMTs). By coupling the recently described Orthogonal Organic Phase Separation (OOPS) strategy with the Stable Isotope Labelling with Amino acids in Cell culture (SILAC) and pharmacological modulation of PRMTs, we profiled RNA-protein interaction dynamics in dependence of protein-R-methylation. Data are available via ProteomeXchange with identifier PXD024601.

## Introduction

Arginine (R)-methylation is a widespread post-translational modification (PTM) that occurs on both histones, where it acts as an epigenetic regulator of gene expression, and non-histone proteins, where it modulates protein-protein, protein-RNA and protein-DNA interactions (Blanc and Richard, 2017), emerging as a key modulator of several cellular processes, from translation and splicing to growth factor–receptor signaling, miRNA biogenesis and DNA damage response (Guccione and Richard, 2019; Musiani et al., 2020; Spadotto et al., 2020). In mammals, nine enzymes have been identified and classified as type I, type II and type III protein R-methyltransferases (PRMTs), depending on their ability to transfer to the guanidino group of the arginine residues either two methyl-groups in asymmetric (ADMA) and symmetric (SDMA) manner, or one methyl-group (MMA), respectively (Blanc and Richard, 2017). Arginines located within glycine-arginine-rich regions, the so called “RGG/RG motifs”, are preferred sites for methylation by PRMTs (Thandapani et al., 2013). In mammals, PRMT1 and PRMT5 are the most active PRMTs of the type I and II families, respectively, and object of intense investigation in both basic and translational research (Zhang et al., 2019). As a matter of fact, various PRMTs have been found overexpressed in several solid tumors -such as breast, lung, colon, bladder, head, neck cancers‐ and hematological malignancies, such as leukemia (Smith et al., 2018; Zhang et al., 2018). Hence, various inhibitors with different selectivity for PRMTs are under development as anti-cancer drugs, some already entering phase-1 and -2 clinical trials (Hu et al., 2016; Kaniskan et al., 2018). In recent years, proteome-wide strategies to study R-methylated proteins have been optimized, thanks to the implementation of efficient biochemical protocols for methyl-peptide enrichment, coupled to off-line high pH (HpH) chromatographic fractionation and high-resolution mass spectrometry (MS) analysis. Recently published evidence (Fedoriw et al., 2019; Fong et al., 2019; Lim et al., 2020; Szewczyk et al., 2020), together with MS-proteomics analyses carried out in our group (Musiani et al., 2019; Musiani et al., 2020; Spadotto et al., 2020), has shown that pharmacological and genetic inhibition of PRMTs coupled with quantitative MS-based analysis are powerful approaches to expand the knowledge about the extent of this modification, its dynamics upon different perturbation and its involvement in different cellular pathways. One interesting piece of information emerging from these studies is that RNA-binding proteins (RBPs) are over-represented among experimentally-annotated R-methylated proteins.

RBPs bind their cognate RNAs either in a sequence-specific manner, through their RNA-binding domains (RBDs), such as the RNA recognition motif (RRM), the hnRNP K homology (KH) domain and the dead/deah box helicase (DDX) domain, or in a structure-dependent fashion, whereby they interact to specific RNA secondary structures rather than nucleotide sequences (Hentze et al., 2018). RBPs are involved in several cellular processes linked to RNA processing, including pre-mRNA splicing, mRNA transport, microRNA biogenesis, and translation; such processes are essential for cell homeostasis and for fine-tuning gene expression in response to perturbations, or during differentiation and developmental transitions, and are frequently dis-regulated in cancer (Yang and Bedford, 2013; Pereira et al., 2017). In addition to the RBDs, these RBPs often contain sequences that have been variously termed as low complexity (LC) region or intrinsically disordered regions (IDRs), which were shown to confer the capability to undergo liquid-liquid phase separation (LLPS) and form membranless organelles (MLOs) (Lin et al., 2015). Notably, these disordered regions very often include RGG/RG motifs, the preferred targets of PRMT enzymes (Chong et al., 2018). This provides strong indication of a mechanistic link between the R-methylation state of RBPs and their capability to undergo LLPS, through a change in their interaction with RNA. In line with this idea, Tsai and colleagues have recently shown that the assembly of stress granules (SGs), a type of cytosolic MLOs, is dependent on the R-methylation level of the SG-nucleating protein G3BP1 (Tsai et al., 2016). Furthermore, FUS protein was shown to undergo phase-separation in the nucleus upon pharmacological inhibition of ADMA by Adenosine Dialdehyde (AdOx), an inhibitor of S-adenosyl-L-homocystein hydrolase that leads to the accumulation of S-adenosyl-L-homocystein (Adoicy), a general inhibitor of methyltransferases (Qamar et al., 2018). This evidence hints towards a more general role of protein-R-methylation in regulating RBP-RNA dynamics and, for some proteins, promoting LLPS. Nevertheless, the mechanistic link between the R-methylation state of a protein and its binding to cognate RNAs has been so far described non-systematically, only for individual cases, while a proteome-wide evaluation is still missing.

To address this question, we carried out the first proteome-wide analysis of global changes of RBP-RNA interactions in dependence of protein R-methylation by applying the Orthogonal Organic Phase Separation (OOPS) strategy (Queiroz et al., 2019) to isolate RBP-RNA complexes and coupling it to Stable Isotope Labelling with Amino acids in Cell culture (SILAC) and pharmacological modulation of PRMTs. The observation that the presence of a subset of RBPs is reproducibly altered in the interface fraction enriched by OOPS upon treatment with PRMT type I (but not type-II) inhibitor suggests that MMA/ADMA levels in these proteins modulate their interaction with RNAs. Moreover, we observed that treatment with the same PRMT inhibitor induces LLPS of some candidate RBPs, whose interaction with RNA was found modulated in the proteomics experiments. Overall, our data confirm that modulation of MMA/ADMA, rather than SDMA, directly impacts on RBP-RNA interactions, with consequent effects on MLO assembly.

## Results

### Analysis of RNA Binding Protein Dynamics in Dependence of PRMTs by SILAC-Based OOPS Strategy

**FIGURE 1:**
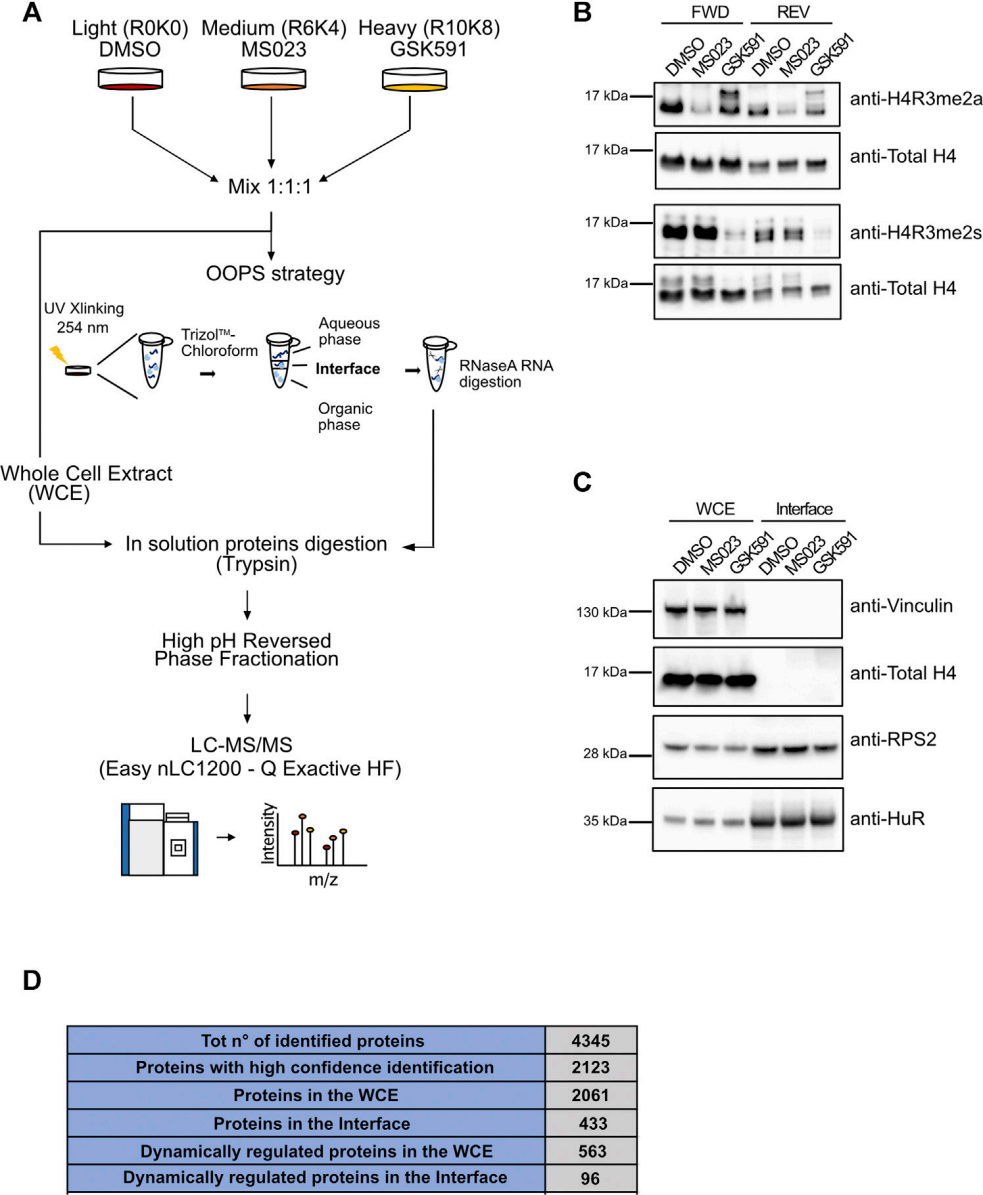
Setup of the proteomic approach for the systematic analysis of RNA-protein interactions: the OOPS strategy in combination with PRMT pharmacological inhibition and triple SILAC labelling. **(A)** Representative workflow of the proteomic approach. Cells were grown in light (R0K0), medium (R6K4) and heavy (R10K8) SILAC medium and treated with DMSO, 10 µM MS023 and 5 µM GSK591 for 48h. Aliquots from the light-, medium- and heavy-labelled cells were mixed in 1:1:1 proportion and saved as whole cell extract (WCE), while the remaining cells were UV-crosslinked at 254 nm and phase-partitioned through a Trizol™ - chloroform mixture, as described in (Queiroz et al., 2019). Proteins extracted from the WCE and from the interface fraction were subjected to in-solution digestion with Trypsin and fractionation by off-line HpH-RP chromatography. Tryptic peptides were analyzed by high resolution LC-MS/MS. **(B)** Western Blot (WB) validation of the PRMT pharmacological inhibition. Before mixing in 1:1:1 proportion described in **(A)**, an aliquot of each condition was used to test the methylation state of distinct histone R residues specifically targeted by PRMT1 and PRMT5 by WB, both in the forward (FWD) and in the reversed (REV) experiment. Reduction of asymmetric di-methylation of arginine 3 on histone 4 (H4R3me2a) was observed upon MS023 treatment; total unmodified H4 was used as loading control. Similarly, the reduction of symmetric di-methylation of arginine 3 on histone 4 (H4R3me2s) was observed upon GSK591 treatment (H4 was used as loading control). **(C)** WB validation of RBPs enrichment by OOPS. WB analysis of the RNA binding proteins RPS2 and HuR confirms their enrichment in the interface fraction upon OOPS, while the absence of the non-RBP protein Vinculin and Histone 4 from the same fraction was used to assess the selectivity of the method. **(D)** Summary of the MS-identified proteins by OOPS. Table summarizing the number of proteins identified by MaxQuant from raw MS data, after the application of the indicated filtering criteria: 1) total number of identified proteins, upon removal of reverse hits and contaminants; 2) total number of proteins with Andromeda score ≥25 and at least two peptides, one of which unique, for each experiment (high-confidence identification); 3) total number of proteins identified with high confidence in the WCE; 4) total number of proteins identified at high confidence in the interface fraction from OOPS; 5) number of proteins dynamically regulated by the drugs in the WCE; 6) number of proteins dynamically regulated by the drugs in the interface fraction from OOPS.

To evaluate the role of protein-R-methylation in the regulation of RBP-RNA interactions, we took advantage of the OOPS strategy to isolate RBP-RNA complexes, coupling it with triple SILAC- proteomics and the use of PRMT inhibitors, in HeLa cervical cancer cells. The experimental design is illustrated in Figure 1A: HeLa cells were grown in light, medium and heavy SILAC culture medium, in order to profile in parallel three conditions: DMSO (as control treatment), PRMT type I and PRMT5 inhibition. The triple SILAC experiment was carried out in two biological replicates, “Forward” and “Reverse”, whereby the medium- and heavy-SILAC channels were swapped among the two drug treatments, to increase the confidence in identification of specific alterations in protein-RNA interactions. Efficient inhibition of PRMTs was achieved with a 48 h treatment with the PRMT type I inhibitor MS023 (which - at the IC50 conditions used in the experiment- mainly targets PRMT1, the most active enzyme in the type I family) (Eram et al., 2016) and GSK591, a selective inhibitor of PRMT5 (Sachamitr et al., 2021). Drug efficiency was confirmed by monitoring changes in ADMA and SDMA, both globally (Supplementary Figure S1) and on the asymmetric di-methylation of Arginine 3 on histone 4 (H4R3me2a) and on the symmetric di-methylation of Arginine 3 on histone 4 (H4R3me2s), modifications known to be specifically deposed by PRMT1 and PRMT5, respectively (Figure 1B). The Western Blot (WB) control of the levels of H3R2me2a, deposed by PRMT6, and of H3R17me2a, set by PRMT4/CARM1, was instead used to confirm the preferential selectivity of MS023 towards PRMT1, at least in the experimental conditions used in this study (Supplementary Figure S1E). Following drug treatment, fractions (about 20%) from the light-, medium- and heavy-labelled cells were mixed in 1:1:1 proportion and saved as whole cell extract (WCE), while the remaining cells were UV-crosslinked at 254 nm and phase-partitioned through incubation with a Trizol™-chloroform mixture, as described in (Queiroz et al., 2019). This step allows separating three fractions: an upper aqueous part containing free RNAs, an interface that contains the RBP-RNA complexes and a lower organic part containing free proteins. For our purpose, we in-depth analysed the interface fraction. To confirm the expected enrichment of RBPs in the interface fraction, we profiled by WB the levels of known RBPs, like RPS2 and HuR, in both the WCE and interface fraction: their enrichment in the interface confirmed the efficiency of the OOPS protocol, while the absence in the same fraction of the non-RNA-binding proteins Histone 4 and Vinculin corroborated its selectivity (Figure 1C). Both WCE and the interface fractions were then selected for subsequent MS-proteomics analysis: proteins were in solution Trypsin-digested and peptides were separated by off-line HpH Reversed Phase (RP) Chromatography prior to Liquid Chromatography-tandem Mass Spectrometry (LC-MS/MS) analysis (Figure 1A). MS analysis on a Q Exactive HF mass spectrometer, followed by data processing for protein identification and quantification with the MaxQuant suite of algorithms (Tyanova et al., 2016), led to the annotation of 2123 proteins identified with at least 2 peptides, one of which unique, and an Andromeda score ≥25. Of them, 2061 were annotated in the WCE and 433 in the interface, respectively (Figure 1D). The majority (425, 98%) of the interface proteins were in common with the WCE, with only 8 proteins exclusively found in this fraction (Supplementary Figure S1H), among which TIAL1, hnRNPD, NOLC1, SPEN and RALY are well-known RBPs.

### Efficiency of the Orthogonal Organic Phase Separation Strategy in Enriching RNA Binding Proteins and Over Representation Analysis of R-Centered Motifs Within the Interface Fraction

**FIGURE 2:**
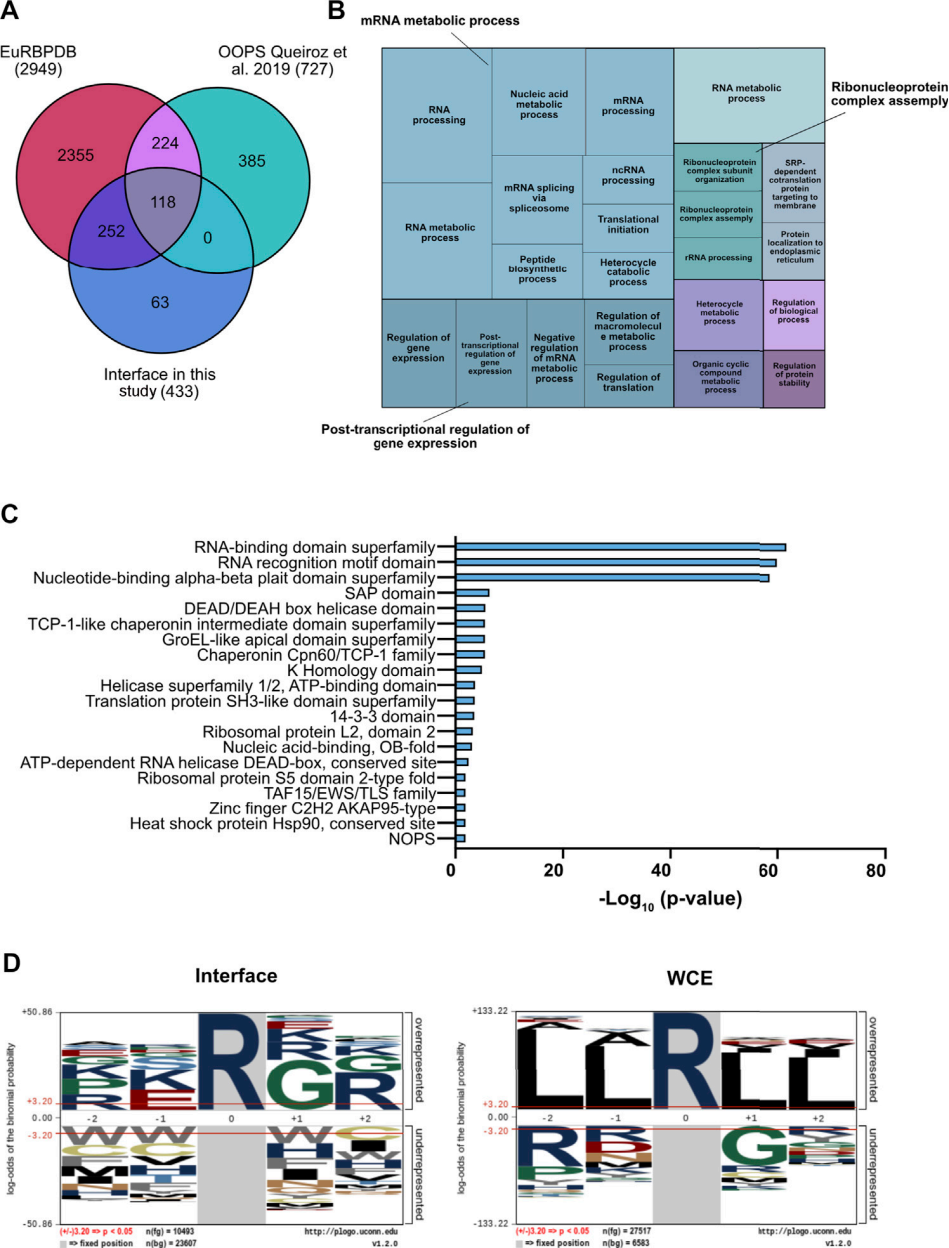
Functional characterization of the interface fraction upon OOPS. **(A)** Comparative validation of RBPs enrichment in the interface fraction. Comparative analysis was performed against the Eukaryotic RNA Binding Proteins Database (EuRBPDB) and the RBPs identified in (Queiroz et al., 2019): 370 out the 433 proteins identified within the interface fraction (85.5%) showed overlap with the other datasets. **(B)** Treemap representation of the GO enriched terms in the interface fraction. GO analysis performed by GOrilla and REVIGO indicates the most enriched GO terms in the interface proteins. **(C)** Domain enrichment analysis of the interface proteins. Analysis was performed by STRING database on the list of the interface proteins **(D)** Over representation analysis of the R-centered sequences. The analysis was performed with the pLogo software, comparing interface and WCE proteins.

To characterize the proteins enriched in the interface fraction upon OOPS, we first compared our experimental list with that annotated by K. Lilley and co-workers, who first optimized the OOPS strategy (Queiroz et al., 2019), and with the EuRBPDB database, a comprehensive repository of eukaryotic RNA-binding proteins (Liao et al., 2020). EuRBPDB includes both “canonical” RBPs containing RBDs and “non-canonical” RBPs that do not contain RBDs but are predicted to bind the secondary structure of cognate RNAs, such as IDRs located in their primary sequences. We found that 370 of the 433 proteins detected in the interface (85%) were validated by the intersection with the two datasets (Figure 2A). Gene Ontology (GO) indicated a good representation of the so-called RNA-binding proteome (RBPome) while the same analysis of the non-overlapping 63 proteins (14.5%) showed that 7 have predicted RNA-binding capability and represent putative novel RBPs, whereas the rest are proteins related to extracellular matrix organization, drug response and phosphorylation-related processes. While we cannot exclude that they may be contaminants, it is also possible that such proteins, while not being intrinsic RNA-binders, are enriched in this fraction through association with genuine RBPs.

The smaller number of proteins enriched in the interface fraction compared to Queiroz et al. could reflect the fact that their dataset was obtained by the combination of data from three different cell lines (HEK293, MCF10A and U2OS) comprising both cancer and non-tumor cells. Functional analysis of our protein list showed an almost exclusive enrichment of biological process related to RNA metabolism, such as RNA splicing, RNA metabolic process, RNA processing and translation (Figure 2B and Supplementary Figure S2C). Moreover, a domain enrichment analysis highlighted the strong over-representation of RBDs and RRMs (*p*-value = e-60), known to be frequently R-methylated (Blackwell and Ceman, 2012; Bedford and Richard, 2005; Fulton et al., 2019). In addition, we found other domains frequently associated to RBPs, such as the SAP domain (Aravind and Koonin, 2000), the DDX (Gilman et al., 2017) and the KH (Valverde et al., 2008) domains, also known to be R-methylated (Figure 2C). The fact that the 14-3-3 protein family was also enriched in the interface fraction is particularly intriguing, because these proteins were the first identified containing a reader motif for phospho-serine/threonine (Espejo et al., 2017); hence this result supports the idea of a possible cross-talk between R-methylation and Ser/Thr-phosphorylation (Chen et al., 2016; Liu et al., 2020; Smith et al., 2020).

Elaborating on the evidence that PRMTs typically recognize and modify arginines located within the glycine-arginine-rich (RG\RGG) domains (Thandapani et al., 2013), we asked whether specific enrichment of such motifs could be observed in an amino acid window around each R located within the proteins annotated in the interface: indeed, the strong enrichment of the RG\RGG domain in the interface proteins, but not in the WCE, corroborates the evidence that RBPs are preferential targets of PRMTs (Figure 2D).

### Pharmacological Modulation of Protein Arginine Methyltransferases Type I, but Not of Protein Arginine Methyltransferase 5, Affects Protein-RNA Interactions

**FIGURE 3:**
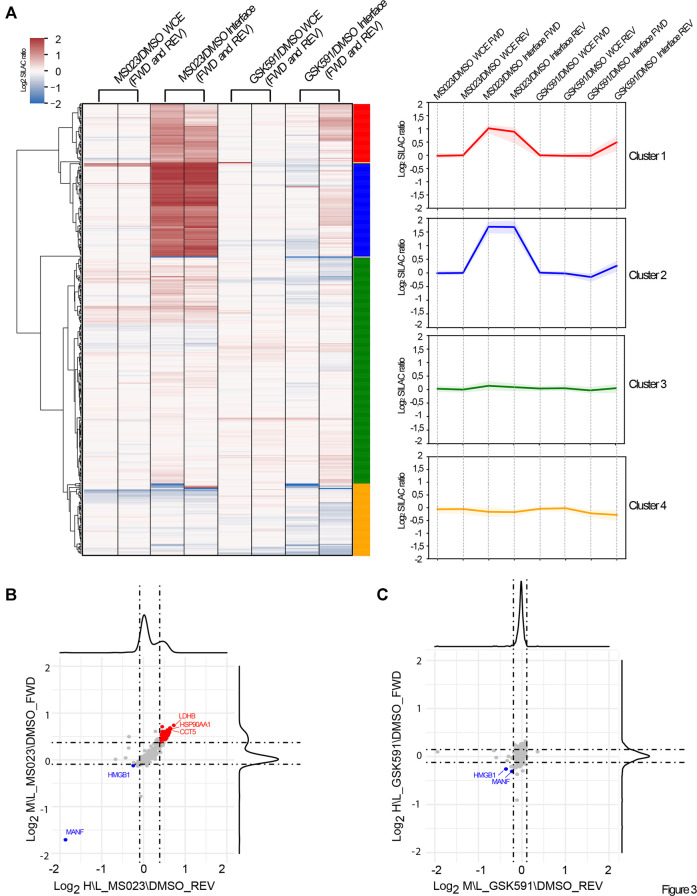
Dynamics of RBP-RNA interactions in dependence of PRMT pharmacological modulation. **(A)** Supervised clustering analysis of the quantitative OOPS proteomics data. Supervised clustering analysis of differential protein expression or differential RNA-binding after MS023 and GSK591 treatment normalized on DMSO led to the identification of four representative clusters: Cluster 1 and Cluster 2 contain proteins with Log_2_ SILAC ratio MS023/DMSO +1 and +1.5, respectively, only in the interface fraction; Cluster 3 and Cluster 4 contains proteins overall not significantly modulated in the interface, with Cluster 4 displaying a mild decrease in the interface upon GSK591 (−0.3 Log_2_ SILAC ratios). **(B)** Scatter plot representation of the normalized Log_2_ SILAC ratio in MS023-treated condition. Scatter plot of the Log_2_ MS023/DMSO SILAC ratio of interface proteins, normalized on the respective protein SILAC ratio in the WCE, in FWD versus REV experiment. Dashed lines indicate μ±σ of the respective SILAC protein ratio distributions; proteins up- or down-regulated are displayed in red and blue, respectively. **(C)** Scatter-plot representation of the normalized Log_2_ SILAC ratio in GSK591 treated condition. The scatter plot displays the Log_2_ GSK591/DMSO SILAC ratio of interface proteins, normalized on the respective SILAC ratio in the WCE in FWD versus REV experiment. Dashed lines indicate μ±σ of the respective SILAC protein ratio distributions; proteins down-regulated are displayed in blue.

Since the majority of RBPs identified by OOPS are putative PRMT targets, we next set to investigate the effect of R-methylation modulation on RBP-RNA interaction. We took advantage of the quantitative information included in our SILAC OOPS experiment coupled with MS023 and GSK591 treatment. We performed supervised clustering analysis of the Log_2_ SILAC protein ratios of the proteins presenting M\L and H\L SILAC ratios (ratio count >0) in all experimental conditions tested, both in the total proteome and in the RBPome. To better highlight changes exclusively affecting RBP-RNA interactions and not protein expression, for each protein we compared the SILAC ratio measured in the interface fraction with the corresponding ratio in the WCE. From 416 proteins profiled upon filtering, four different clusters emerged (Figure 3A), which reflect either the different protein expression or association with cognate RNAs, upon modulation of PRMT type I and PRMT5: Cluster 1, including 53 proteins (red), and Cluster 2, including 85 proteins (blue), represent proteins that show increased levels in the interface fraction (+1 and +1.5 Log_2_ SILAC ratio, respectively), but not in the WCE upon MS023 treatment, with no significant changes upon GSK591. This pattern indicates that MS023 has a positive impact on the interaction of these proteins to RNAs and that this increase is not a mere consequence of protein expression up-regulation. Cluster 3, including 206 proteins (green), and Cluster 4, including 65 proteins (yellow), represent proteins that are overall not changing after pharmacological treatment, with a minor down-regulation observed in Cluster 4. Too few to belong to any specific cluster, we identified MANF and HMGB1, whose level is however significantly down-regulated (-5 and -1 Log_2_ SILAC ratio, respectively) in the interface fraction, but not in the WCE, upon both MS023 and GSK591 treatment. This readout suggests that the altered R-methylation level of these two proteins reduces their binding to RNA, with no effect on protein expression. By plotting the MS023\DMSO SILAC ratio of the interface proteins (normalized over the corresponding SILAC ratio in the WCE) in the forward versus the reverse experiment and defining the significant outliers based on the SILAC protein ratio distributions, we identified 76 proteins up-regulated (+1σ) and 4 down-regulated (−1σ) upon MS023 (Figure 3B); the same analysis carried out with the GSK591\DMSO SILAC ratio led to the identification of only 4 proteins significantly down-regulated (−1σ), of which 2 were also down-regulated by MS023; no proteins appeared to be up-regulated in the interface fraction with this drug (Figure 3C).

Taken together, these results indicate that inhibition of PRMTs type I has a much stronger impact on RBP-RNA interactions than PRMT5 blockage and they corroborate the hypothesis that alteration of ADMA\MMA levels of a set of proteins could be directly involved in the modulation of their interaction with cognate RNAs.

### Validation of MS-Proteomics Data Confirms That RNA Interaction of a RBP Subset Is Modulated by Protein Arginine Methyltransferase 1 Inhibition

**FIGURE 4:**
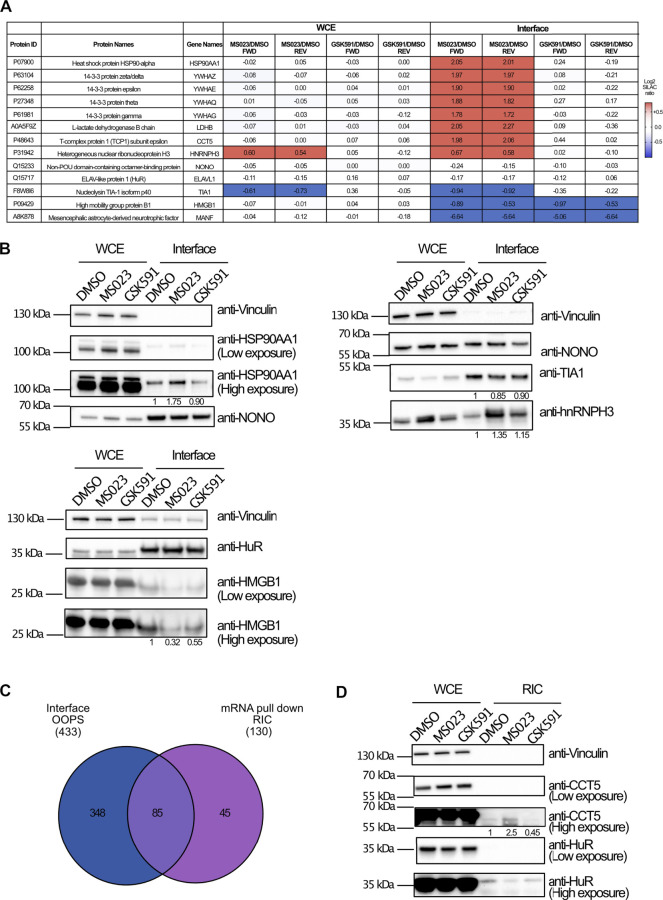
WB validation of the MS-proteomics data. **(A)** Overview of representative RBPs quantified by SILAC OOPS. Table summarizes the MS023/DMSO and GSK591/DMSO SILAC protein ratio of representative proteins, both in the WCE and in the interface fraction, both in FWD and REV experiment. **(B)** WB validation of the differential protein response to PRMT inhibitors upon OOPS. WB profiling of representative proteins, whose MS023/DMSO SILAC protein ratio is summarized in **(A)**, was used to assess the different modulation upon drugs treatment in both WCE and interface fraction: HSP90AA1 and HMGB1 were selected as examples of proteins up-regulated and down-regulated, respectively, in the interface fraction but not in WCE upon MS023; hnRNPH3 was selected as example of protein up-regulated upon MS023 in the interface as consequence of a similar modulation in the WCE; TIA1 was selected as example of protein down-regulated upon MS023 treatment as consequence of a similar modulation in the WCE; NONO and HuR, displaying SILAC protein ratios around 1 in the interface, were selected as loading controls for the interface fraction. Protein abundance in the interface upon different treatments were evaluated upon multiple normalization of band intensities, as described in the *Materials and Methods* section. **(C)** Comparative analysis of proteins identified by OOPS and by RIC-MS experiment. Intersection of the proteins in the interface fraction from OOPS and those identified by RIC-MS allows validating 85 proteins identified in both experiments upon stringent filtering of MS-data (Andromeda score ≥25, at least 2 peptides identified per protein, one of which unique, for each experiment). **(D)** Western Blot validation of differential protein response to MS023 in the RIC experiment. WB analysis of CCT5 and HMGB1 protein upon MS023 treatment in the RIC experiment confirms their increased and decreased binding to RNA, respectively. Vinculin and HuR were used as loading control for the WCE and the interface, respectively. Protein abundances in the RNA pull-down fraction upon different treatments were evaluated upon multiple normalization of band intensities, as described in the *Materials and Methods* section.

The proteomics data revealed different protein responses in terms of interaction with RNAs, with more pronounced changes upon PRMT type I inhibition, which affects globally ADMA/MMA balance. We selected some proteins representative of these different responses to validate the SILAC data by WB analysis. NONO and HuR proteins, whose SILAC ratios were unchanged in all fractions upon the two drugs, were profiled as representative of the RBPs whose interaction with cognate RNAs is R-methylation independent (Figure 4A); HSP90AA1 and HMGB1 belong to the subset of proteins with significantly modulated (up- and down-regulated, respectively) SILAC ratio in the interface fraction upon treatment with MS023, which was not reflected in the WCE (Figure 4A). The WB analysis confirmed their altered levels in the interface fraction when normalized on NONO and HuR levels in the corresponding functional states (Figure 4B). On the other hand, hnRNPH3 and TIA1 were selected as examples of proteins whose altered levels in the interface upon drug treatment followed expression changes in the WCE. In particular, hnRNPH3 was up-regulated by MS023 both in WCE and in interface, while TIA1 resulted down-regulated in both fractions (Figure 4B). Hence, even if PRMT modulation could partly affect their RNA-protein capability, this change seems mainly a reflecion of their altered expression (Figure 4B).

To confirm the more prominent involvement of PRMT1 in governing these dynamics, we used OOPS-WB analysis in HeLa cells which were depleted of PRMT1 upon transfection with two distinct shRNA constructs and a scrambled shRNA, as negative control. OOPS was carried out and selected proteins were WB-profiled in wild-type and PRMT1 KD conditions, both in WCE and interface fraction: the observation of the specific increase of HSP90AA1 and decrease of HMGB1 in the interface when PRMT1 was depleted confirmed the effect observed upon treatment with MS023 and corroborated the OOPS-MS data (Supplementary Figures S3A,B).

The OOPS experiment coupled with SILAC and external perturbation allows to enrich for RBPs associated to their cognate RNAs and to assess their dynamic behaviour. This experiment can be used to infer alterations of specific protein-RNA interactions, however, an important limitation is the lack of direct proof of changes in binding of individual proteins with the respective RNA partners. To address this point and corroborate the OOPS-MS data with a complementary method, we performed the RNA Interactome Capture (RIC) experiment, which enables to pull-down poly(A)-RNAs by oligo(dT)-conjugated beads and the co-associated proteins, which are then identified by MS (Castello et al., 2013; Perez-Perri et al., 2018). The RIC approach is complementary to OOPS because it is based on affinity-enrichment and direct protein-mRNA interaction, while OOPS is based on a biochemical fractionation strategy that allows analysing proteins associated also to non-polyadenylated RNAs.

We coupled RIC with triple SILAC labelling upon pharmacological inhibition of PRMT1 and PRMT5. Upon RNA pull-down, protein extraction, digestion, LC-MS/MS analysis and MaxQuant processing of the MS data, we produced a list of 130 RBPs identified in at least one of the two replicates, in the different conditions. Protein SILAC ratios in the RNA-pulldown fraction were normalized over the corresponding SILAC ratios in the WCE used as input, in order to distinguish genuine changes in protein-RNA interactions from mere protein expression alterations (Supplementary Table S2).

When the proteins annotated at the interface from OOPS were intersected with the protein list from RIC, we found a rather limited overlap (Figure 4C), with 18% of the OOPS proteins also identified in RIC, whose dataset was much smaller. The limited overlap and the dissimilar size of the two proteomes can be explained in light of the different rationale and biochemical procedure for putative RBP enrichment, whereby RIC enrichement is limited to messenger RNAs while OOPS allow fractionating a broader spectrum of RNAs and the associated proteins, as also reflected by the GO analysis carried out on the two proteomes (Supplementary Figure S3C). Despite the limited overlap, we focused on the ratios of the proteins in common: a good proportion of proteins (61 out of 85, corresponding to 71% of the RIC dataset), comprising the hnRNP family proteins, the ribosomal proteins RPS2 and RPS10, NONO and HuR, resulted unchanged both in OOPS and RIC. More interestingly, proteins displaying a reduced level in the OOPS fraction upon MS023, such as MANF and HMGB1, were also found down-regulated in the RIC experiment. Unfortunately, no proteins up-regulated in the OOPS were detected by RIC, so their dynamic behavior could not be validated. To be more explorative and expand the overlap between the OOPS and RIC datasets, we relaxed the filtering criteria applied and considered as valid hits all proteins identified in at least one of the two replicates, removed the Andromeda score>25 and the criterium that, for each protein, the SILAC ratio should be measured both in the interface/RIC and WCE, for normalization. The intersection from these relaxed datasets led to a higher number of proteins in common, from 85 to 108 (Supplementary Figure S3D). Among them, the majority (75%) resulted not significantly changed neither in the RIC nor in the OOPS experiments upon drug treatment; the group of significantly down-regulated protein was enriched with 9 proteins (TCEA1, NQO1, HISTH1E, RPL26, RPL7A, RPS27A, RRBP1, H2AFV and FKBP3) in addition to MANF and HMGB1. More importantly, we found the protein RALY, whose dynamic increase upon MS023 was observed in both experiments (Supplementary Table S2). As a final confirmation of our results, at least for the protein CCT5 that was up-regulated in the interface upon MS023 but not identified in the RIC-MS experiment, we carried out the WB profiling upon RIC in untreated and drug-treated cells, confirming its increased association with RNA upon type I PRMT inhibition, while the HuR stable behavior served as an additional validation (Figure 4D).

Despite the restrains linked to the limited overlap between the two complementary methods, these results support our working hypothesis that -at least for a subset of proteins - the modulation of PRMT1 causes their altered interaction with cognate RNAs, probably through a change of their ADMA\MMA modification level.

### RNA Binding Proteins-RNA Dynamics Is Linked to Changes in the Asymmetric-R-Methylation State of RNA Binding Proteins

**FIGURE 5:**
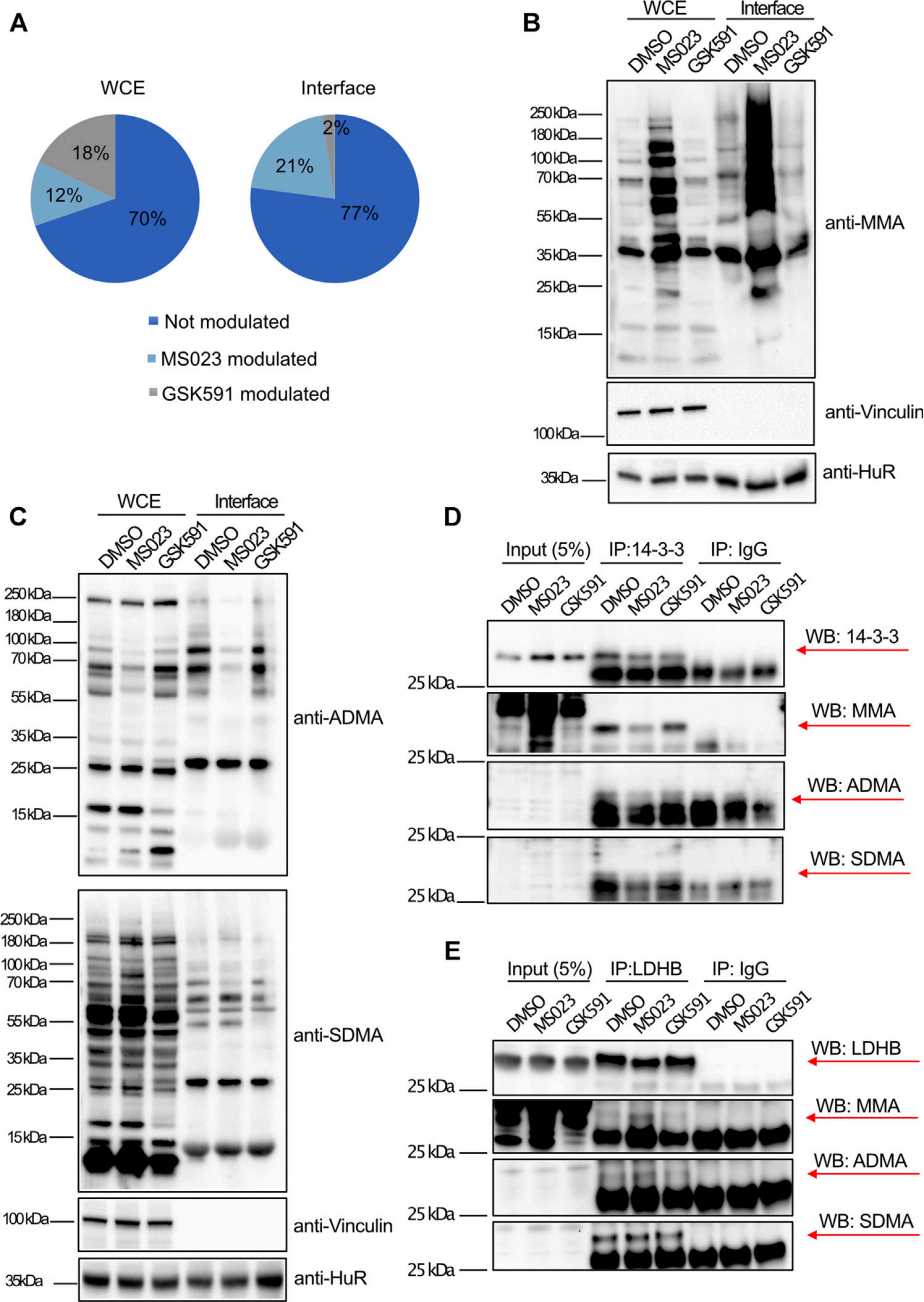
Interface-enriched protein dynamic binding to RNA is associated to their R-methylation state. **(A)** Percentage of protein modulated in response to PRMT inhibitors. Percentage of protein significantly modulated (±1σ) was calculated in dependence of MS023 or GSK591 treatment, both in WCE and interface fraction. In the WCE the two treatments equally modulate protein expression (12% regulated by MS023 and 18% by GSK591, respectively), whereas in the interface fraction, RBP-RNA interactions are almost exclusively regulated by MS023 treatment (21% regulated by MS023 and 2% regulated by GSK591, respectively). **(B)** WB profiling of dynamic regulation of global protein R-mono-methylation (MMA). WB was carried out on aliquots of WCE and interface fraction in control DMSO and upon MS023 and GSK591. Vinculin and HuR protein were used as loading control for WCE and interface, respectively. **(C)** WB profiling of dynamic regulation of protein asymmetric R-di-methylation (ADMA) and symmetric R-di-methylation (SDMA). WB analysis was carried out on aliquots of WCE and interface fraction in control DMSO and upon MS023 and GSK59. The same membrane was first probed with anti-ADMA antibody, then stripped and used to detect SDMA. Vinculin and HuR protein were used as loading control for WCE and interface, respectively. **(D)** Protein immunoprecipitation (IP) followed by WB validation of the R-methylation state of 14-3-3 proteins as representative for MS023-modulated RBPs in the OOPS. The R-methylation states of 14-3-3 protein was assessed upon DMSO, MS023 or GSK591 treatment by protein IP followed by probing with the anti-pan-methyls antibodies against MMA ADMA and SDMA. IgG were used as mock controls for IP. For 14-3-3 proteins MMA is clearly detectable, while ADMA and SDMA signals are ambiguous, due to the cross-contaminating signals of the light chains of denatured antibodies. **(E)** Protein IP followed by WB validation of the R-methylation state of LDHB protein as representative for MS023-modulated RBPs in the OOPS. The R-methylation states of LDHB protein was assessed upon DMSO, MS023 or GSK591 treatment by protein IP followed by probing with anti-pan-methyl antibodies against MMA ADMA and SDMA. IgG was used as mock control for IP. MMA and SDMA are clearly detectable, while the ADMA signal is ambiguous, due to the cross-contaminating signals of the light chains of denatured antibodies.

To understand whether the changes in protein-RNA interactions observed in Cluster 1 and 2 could be linked to possible alteration in the protein R-methylation state, we compared the percentage of protein dynamically modulated by the two inhibitors in the WCE and in the interface fraction: only 12% of the whole proteome is modulated by MS023 while this fraction increases to 21% in the interface fraction (Figure 5A). Fisher’s exact test applied to these percentages confirmed that the fraction of modulated proteins in the interface is statistically significant (*p* < 0.0001); so, modulation of MMA/ADMA levels seems to affect protein-RNA interactions, beyond mere gene expression effects. Such difference could not be detected when using GSK591, where we even observed a reduction in the proportion of protein modulated by GSK591 in the interface fraction compared to the WCE (2% versus 18%, respectively). Following the same reasoning, we carried out WB profiling of global protein-R-methylation upon MS023 and GSK591 in WCE and interface fraction using pan-antibodies against ADMA, SDMA and MMA. In the WCE, we detected a stronger effect induced by MS023 than by GSK591, measured by an overall stronger reduction of ADMA than SDMA (Figure 5C). As previously observed (Eram et al., 2016), inhibition of PRMT type I by MS023 led also to increased MMA (Figure 5B) that paralleled ADMA reduction; this can be interpreted as the result of the substrate scavenging effect by other enzymes when PRMT1 is blocked (Dhar et al., 2013) (Figure 5B). Interestingly, overall changes in global ADMA, SDMA and MMA upon the two drugs were more marked in the interface fraction than in the WCE, which -in our opinion- indicates that RBPs enriched in this fraction are overall more R-methylated and that their R-methylation state is more modulated.

We then asked how many of the proteins regulated by MS023 in the OOPS experiment are annotated as R-methylated, using as experimental references both the protein post-translational modification database PhosphositePlus (Hornbeck et al., 2015) and our in-house experimentally annotated high-confidence methyl-proteome (*manuscript in preparation*): 51 out of 103 (49.5%) proteins modulated in the WCE and 59 out of 77 proteins (76.6%) modulated in the interface fraction, respectively, are annotated as R-methylated (Supplementary Figure S3A). Fisher’s exact test calculated on these percentages confirmed that the enrichment of R-methyl-proteins within the interface modulated proteins is statistically significant (*p* < 0.0001), which supports the idea that the changes in the RBP-RNA interaction observed are *bona fide* mechanistically linked to their R-methylation state. To validate the R-methylation state of representative RBPs, we performed the immunoprecipitation (IP) of 14-3-3 and LDHB proteins followed by probing their R-methylation state with pan-antibodies against ADMA, SDMA and MMA. In the PhosphositePlus database, 14-3-3 and LDHB proteins are annotated as R-monomethylated, and indeed MMA of both proteins resulted modulated upon MS023 treatment, in line with our proteomics evidence from OOPS, that was interpreted as an altered interaction with RNA, whereas the detection of asymmetric and symmetric R-di-methylation was ambiguous or unchanging upon drug treatments (Figures 5D,E). Overall, these data indicate that, at least for the set of proteins inspected, the observed change in their RNA-interaction is linked to an alteration of their R-methylation, triggered by PRMT pharmacological inhibition.

### Modulation of the ADMA/MMA Balance but Not of SDMA Induces Phase-Separation of Candidate RNA Binding Proteins

**FIGURE 6:**
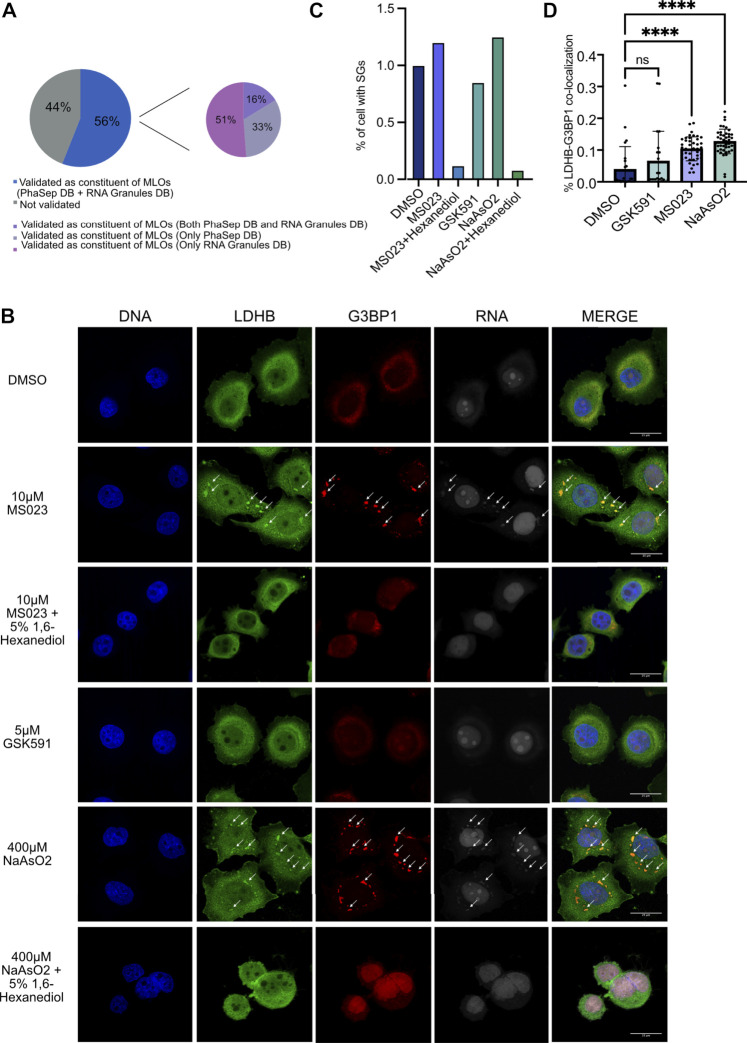
MS023 treatment induces LLPS of candidate RBPs. **(A)** Tendency of MS023-modulated RBPs to undergo LLPS. Among the 77 proteins up-regulated in the interface fraction upon MS023, 43 (56%) were also annotated as proteins undergoing phase separation in at least one of the two PhaSepDB (http://db.phasep.pro/) and RNA Granules DB (http://rnagranuledb.lunenfeld.ca/) databases. Among them, 16% were annotated in both databases, 33% were annotated only in PhaSepDB and 51% were annotated only in RNA Granules DB. **(B)** Immunofluorescence (IF) analysis of LDHB in basal condition (DMSO) and in response to different treatments. Representative IF image shows LDHB protein subcellular localization in HeLa cells treated with the following compounds: DMSO, 10 μM MS023 and 5 μM GSK591 for 48 h; 10 μM MS023 for 48 h, followed by 10 min treatment with 5% 1,6-Hexanediol; 400 µM NaAsO_2_ for 30 min, or 400 µM NaAsO_2_ for 30 min followed by 10 min-treatment with 5% 1,6-Hexanediol. Immunostaining of RNA was performed with the Click-iT™ RNA Alexa Fluor™ 594 Imaging Kit. DAPI staining was used for nuclei visualization (DNA). G3BP1 staining was used as positive control for SGs formation. DAPI, LDHB, G3BP1 and RNA staining and the respective merged images are displayed. Images were taken by SP8 confocal microscopy using a 60× oil objective, and a scale bar of 25 μM are included in the merged figure. Arrows indicate co-localization of target RBP, G3BP1 and RNA. **(C)** Bar-graph representation of the percentage of cells with stress granules. The bar-graph shows the percentage of cells with at least 1 G3BP1-positive stress granule (SG) for the different conditions; all the treatments were normalized over DMSO. **(D)** Bar-graph representation of the percentage of LDHB-G3BP1 co-localization. The image describes the percentage of co-localization between G3BP1 and LDHB in the SGs in each condition. All the treatment were normalized over the DMSO. Statistical significance was calculated by Student’t Test (* = *p* <0.05).

Based on the data acquired and in light of published evidence that reduction of ADMA can induce changes in the subcellular localization of some RBPs and-in some cases-also their aggregation and LLPS, we intersected the list of proteins dynamically regulated in the interface fraction upon MS023 with two available databases of proteins undergoing LLPS: the Phase Separation Database (PhaSepDB) (You et al., 2020) and the RNA Granule Database (http://rnagranuledb.lunenfeld.ca/). This comparison revealed that 42 out of 77 (54.5%) proteins are indeed annotated as capable to phase-separate (Figure 6A). Among them, we selected LDHB, 14-3-3 proteins and CDC37 to verify their capability of undergoing phase-separation in response to MS023 treatment. To do so, we followed their subcellular localization by immuno-fluorescence (IF) analysis and assessed their co-localization with both cognate RNAs and the SGs marker G3BP1 (Yang et al., 2020; Wheeler et al., 2016). While proteins were IF-profiled by antibodies, RNA was labelled using the “click” chemistry strategy to incorporate the uridine analog 5-ethynyluridine (EU) into RNA from differentially treated cells, so that EU-labelled RNA could be detected by IF (Jao and Salic, 2008). IF analysis showed that MS023 treatment induces cytosolic aggregates in which the proteins under investigation co-localize with both G3BP1 and EU-labelled RNA (Figure 6B and Supplementary Figure S4A, respectively); such aggregates were overall not observed (or detected to a much lower extenet) when cells were treated with either DMSO or GSK591. Remarkably, MS023-induced granules were disassembled upon treatment with 1,6-Hexanediol, an alcohol widely used for solubilisation of MLOs (Duster et al., 2021), which confirms the phase-separation origin of these RNPs. As further control, we also profiled the subcellular localization of LDHB and 14-3-3 proteins upon NaAsO_2_, a compound well-known to induce SGs formation: as expected, stronger and more numerous G3BP1-stained MLOs were formed upon NaAsO_2_ treament, which remarkably displayed co-localization with our proteins of interest and RNA. Also in this case, the disassembly of such granules by 1,6-Hexanediol confirmed their nature as MLOs.

Unbiased and automatic quantification analysis of IF images demostrated an increase of the percentage of cells displaying at least one of these RNP granules per cell, both in MS023 and NaAsO_2_ conditions, and that such granules are completely abolished upon incubation with 1,6-Hexanediol (Figure 6C and Supplementary Figure S4A). Morevoer, a statistically significant increase of co-localization percentage of the candidate RBPs with G3BP1 in the granules was measured after MS023 and NaAsO_2_ compared to DMSO; GSK59 instead did not lead to such increase of co-localising granules.

Collectively, these results corroborate our hypothesis that the MS023-triggered alteration of R-methylation state of specific RBPs leads to their increased interaction with cognate RNAs which-in turn-favors their tendency to undergo LLPS and generate MLOs.

## Discussion

Through a quantitative proteomics approach, we have described that modulation of protein-R-methylation, and in particular of ADMA/MMA, can affect protein-RNA interactions and that this process is linked to the capability of some RBPs to undergo LLPS. The pharmacological modulation of PRMTs type I was achieved by treating cells with the small molecule MS023, whereas the selective inhibition of PRMT5 was obtained using GSK591 compound. It is generally accepted that PRMT1 is the most active among the type I family and that PRMT1 is the most inhibited enzyme at the concentrations of MS023 used in this study, as also confirmed by the unchanged levels of H3R2me2a and H3R17me2a, known targets of PRMT6 and PRMT4/CARM1, respectively, observed upon drug treatment (Supplementary Figure S1E). Obviously, while we cannot completely rule out the involvement in the regulation of RBP-RNA interaction dynamics of other members of the PRMTs type I family-such as PRMT2, PRMT3, and PRMT8- the observation that PRMT1 knock-down recapitulates the molecular effect observed upon MS023 is a futher corroboration of the key role played by this enzyme (Supplementary Figures S3A,B). However, substrate scavenging has also been observed among different PRMTs, in particular when PRMT1 is blocked, with consequent release of its preferential target sites (Dhar et al., 2013). Hence, more systematic studies will be needed to understand whether other enzymes of the family are involed in this specific cellular process.

In the last years, several biochemical strategies have been introduced for RBP-RNA complexes enrichment, which can be classified in two main groups: RNA-centric and protein-centric strategies (Ramanathan et al., 2019). The former group of methods includes RNA immunoprecipitation (RIP), RNA interactome capture (RIC) (Perez-Perri et al., 2018), RNA interactome using click chemistry (RICK) (Bao et al., 2018), click chemistry-assisted RNA interactome capture (CARIC) (Huang R. et al., 2018) and cross-linking and immunoprecipitation (CLIP) approach, with its variants (HITS-CLIP, iCLIP, eCLIP and PAR-CLIP) (Ule et al., 2018). The majority of these methods includes a step of poly(A)-RNAs capture via hybridization to oligo(dT) beads under denaturing conditions, with proteins directly bound to poly(A)-RNAs co-enriched and then identified by MS. This limits these approaches to poly(A)-RNAs, excluding bacterial or eukaryotic non-polyadenylated RNAs, as also observed in our study, where we detected a sensibly smaller number of proteins more strongly associated to functional processes linked to mRNAs than what obtained by OOPS (Figure 4C and Supplementary Figure S3C). Moreover, these strategies are difficult to scale up for system-wide RBPs analyses and for multiplexed profiling in dynamic conditions. The latter group comprises protein-centric methods that are based on the biochemical separation of RNP complexes using the principle of chemical phase partition, where RNAs and proteins are physically co-enriched and then separately analyzed by RNA-seq and LC-MS/MS, respectively. Among them, three very similar methods have been described: XRNAX (Trendel et al., 2019), PTex (Urdaneta et al., 2019) and OOPS; the last one was adopted in this study and coupled with PRMT inhibitor treatment to assess RNPs dynamics in dependence of protein-R-methylation. We slightly modified the published biochemical workflow of the OOPS to include a step of HpH-RP-chromatography after tryptic digestion of the interface-enriched proteins and prior to MS analysis. The efficacy of the HpH-RP-fractionation was assessed during the optimization phase of the experiment, by comparing the number of proteins identified from the interface, with or without the introduction of this step (Supplementary Figure S1G). While its introduction led to a significant increase of the protein identification rate, most likely by simply increasing peptide separation prior to MS detection, clearly it did not outcompete the number of proteins identified by (Queiroz et al., 2019), in which multiple experiments were pooled to generate the reference dataset.

The comparison of our experimental list of interface proteins annotated by OOPS with the EuRBPDB (Liao et al., 2020) and the Quieroz et al. datasets indicates 85.5% proteins in common, so that we can *bona fide* state that our dataset is a good representation of the known RBPome. Among the 14.5% non-overlapping proteins, we found essentially three protein categories by GO analysis: 1) a set of proteins recognized as RNA-binding, or somehow related to RNA-based process, such as CDK11A, GNB2L1 and ATP5A1; they are *bona fide* novel RBPs that could be added to the RBPome databases; 2) a group of “structural” and highly abundant proteins -such as TUBB8, LAMB1 and ACTC1- which are probable contaminants; 3) a set of proteins related to protein-phosphorylation which could be functionally linked to R-methylation because of the known crosstalk between these two PTMs (Chen et al., 2016; Liu et al., 2020; Smith et al., 2020). Domain enrichment analysis of the protein found in the interface fraction confirms the over representation of several RBDs: the RRM, the DDX and the KH domains. Sequence analysis of the interface proteins confirmed that RGG motif is enriched in this fraction, in line with data describing RG\RGG domains as preferred substrate recognition motifs for PRMTs and able to promote RNA binding (Castello et al., 2016), which set foundations to the hypothesis underlying this study. Clustering analysis of the experimental proteomics data allow quantifying a protein subset in the interface fraction significantly enriched upon MS023 treatment but with no changes at the expression level: remarkably, >75% of them resulted as R-methylated upon intersection with a datasets of experimentally-validated methyl-proteins (Supplementary Figure S3A). While the WB validation confirmed the MS-data, it is important to keep in mind that SILAC-MS analysis provides a much more accurate quantification of proteins than WB; hence, minor variations in proteins levels among different functional states detected by proteomics could be missed by antibody-based approaches.

A protein-protein interaction analysis by Cytoscape (https://cytoscape.org/) carried out on the MS023-regulated RBPs from the interface produced a high-density network in which each protein interacts at least with another partner within the same group (Supplementary Figure S3B). Interestingly, the RBPs with higher node degrees, such as HSP90AA1, HSP90AB1, YWHAE or TCP1, were also those displaying the higher SILAC ratios upon MS023 treatment. These results suggest that -by applying the OOPS protocol- we may enrich not only for proteins directly interacting with RNAs, but also for some of their co-interactors: for instance, HSP90AA1, HSP90AB1, and CDC37 are reported to belong to the same pathway and were shown to physically interact (Zuehlke et al., 2015; Haase and Fitze, 2016). Upon proteomics analysis, we experimentally demonstrated that LDHB, CDC37 and 14-3-3 proteins (RBPs whose association with RNA is MS023-dependent) are also capable to form MLOs and co-localize with G3BP1 and RNA into RNPs, under pharmacological treatment. This evidence of a link between protein-R-methylation, RNP dynamics and LLPs is not surprising in light of the fact that the majority of proteins found in the interface are enriched in RGG/RG motifs, which are over-represented in disordered regions and contribute to conferring phase-separating capability (Chau et al., 2016; Huang L. et al., 2018; Chong et al., 2018; Qamar et al., 2018; Mersaoui et al., 2019; Huang et al., 2020; Kawahara et al., 2021). It is however, notable that our proteomics approach suggested novel candidate proteins involved in this physicochemical process. Interestingly, MLOs have been recently suggested to play roles in cancer chemo-resistance (Loll-Krippleber and Brown, 2017; El-Naggar and Sorensen, 2018; Zhan et al., 2020). An attractive perspective is to investigate wether they can be targeted to counteract tumor chemo-resistance, by impairing their capability to undergo LLPs and form MLOs.

## Materials and Methods

### Cell Culturing and Stable Isotope Labelling with Amino acids in Cell Culture Labelling

HeLa cells were cultured in Dulbecco’s modified Eagle’s medium (DMEM) already including 1% glutamine and supplemented with 10% fetal bovine serum (FBS; Life Technologies), penicillin (100 U/ml), and streptomycin (100 mg/ml). Cells were cultured at 37°C in a 5% CO2 humidified atmosphere. The cells were tested free of mycoplasma contamination. MS023 was purchased from Cayman chemicals; GSK591 was purchased from Sigma Aldrich. Both MS023 (10 µM) and GSK591 (5 µM) were used for 48 h treatment, together with DMSO as control. For triple SILAC, HeLa were grown in ‘‘Light’’, ‘‘Medium’’ and ‘‘Heavy’’ SILAC DMEM (Thermo Fisher Scientific), supplemented with either L-Arginine, L-Lysine or their medium (Arg6: Sigma-Aldrich; Lys4: Sigma-Aldrich) or heavy (Arg10: Sigma-Aldrich; Lys8: Sigma-Aldrich) isotope-counterparts. Arginine and Lysine were added at a concentration of 84 mg/L and 146 mg/L, respectively. SILAC media were supplemented with 10% dialyzed FBS (GIBCO, Life Technologies), 100 U/ml Penicillin and 100 mg/ml Streptomycin. HeLa cells were grown in the respective heavy-isotopes containing media for at least 9 replication cycles, to ensure full incorporation of isotope-encoded amino acids, with a careful monitoring of growth rate, viability and overall morphology, to guarantee that normal cell physiology was preserved.

### Orthogonal Organic Phase Separation Experiment

The OOPS protocol was applied as described in (Queiroz et al., 2019), with adjustments based on the specific biological question to be addressed. Briefly, HeLa cells were exposed to UV (254 nm) at a dose of 40 J/m2 using a Stratalinker 2400 UV cross-linker (Stratagene, La Jolla, CA). Immediately after crosslinking, cells were scraped in 1 ml Trizol™(Fisher Molecular Biology) and transferred to an Eppendorf tube; for biphasic extraction, 200 μl of chloroform (Fisher Scientific) were added, phases were vortexed and centrifuged for 15 min at 12,000 x g at 4°C. The lower organic phase (containing non-crosslinked proteins) was transferred to a new Eppendorf tube and proteins precipitated by addition of 9 volumes of propan-2-ol (Fisher Scientific). The interface fraction (containing the Protein-RNA complexes) was subjected to two additional phase separation cycles and precipitated by addition of 9 volumes of propan-2-ol. The precipitated interface fraction was resuspended in 100 μl of RNA digestion buffer (100 mM TEAB, 1 mM MgCl_2_, 1% SDS) incubated at 95°C for 20 min, cooled down and digested with 4 μg RNase A, incubating overnight at 37°C. The following day, after a final Trizol™-chloroform phase partition, proteins in the organic phase were precipitated by addition of 9 volumes of propan-2-ol and resuspended in urea lysis buffer (9 M urea, 20 mM Hepes (pH 8.0)).

### Protein Sample Preparation Prior to MS and LC-MS/MS Analysis

Two independent SILAC based-proteomics experiments were carried out with swapped SILAC channels (Forward and Reverse experiments), both for the whole cell extracts and the interface fractions. For each SILAC experiment, equal numbers of light-, medium- and heavy-labeled HeLa cells differentially treated with either DMSO or PRMT inhibitors were mixed in a 1:1:1 ratio, pelleted and washed twice with PBS. For preparation of the WCE, cell pellets were lysed in urea lysis buffer (9 M urea, 20 mM Hepes (pH 8.0)) supplemented with 1× protease and phosphatase inhibitors cocktail (Roche), sonicated and cleared by ultracentrifugation (20,000g for 15 min at 15°C). For the RBPome, the protein extract was already resuspended in Urea lysis buffer, following OOPS strategy. For in-solution digestion, 200 µg of proteins were reduced by adding 4.5 mM dithiothreitol (DTT) (Sigma-Aldrich) for 30 min at 55°C, alkylated with 5.5 mM iodoacetamide (10% (v/v) for 15 min at RT in the dark; Sigma-Aldrich), and digested overnight with sequencing-grade trypsin (1:100 (w/w); Promega) after a four-fold dilution in 25 mM ammonium bicarbonate solution. Protease digestion was terminated by the addition of 1% trifluoroacetic acid (TFA) to adjust pH < 3. Debris was removed by centrifugation for 15 min at 1780g at RT. Peptides were dried with a vacuum concentrator, re-suspended into 300 µl of 0.1% TFA and off-line High pH fractionated by Pierce™ High pH Reversed-Phase Peptide Fractionation Kit (Thermo Fisher scientific). Eluted fractions were dried with vacuum concentrator and resuspended in an aqueous 0.1% TFA solution prior to analysis by LC-MS/MS.

### Nano-LC-MS/MS Analysis

Peptide mixtures were analyzed by online nano-flow LC-MS/MS using an EASY-nLC 1000 (Thermo Fisher Scientific, Odense, Denmark) connected to a Q Exactive instrument (Thermo Fisher Scientific) through a nano-electrospray ion source. The nano-LC system was operated in one column set-up with a 50-cm analytical column (75 mm inner diameter, 350-mm outer diameter) packed with C18 resin (EasySpray PEPMAP RSLC C18 2M 50 cm × 75 M, Thermo Fisher Scientific) configuration. Solvent A was 0.1% formic acid (FA) and solvent B was 0.1% FA in 80% ACN. Samples were injected in an aqueous 0.1% TFA solution at a flow rate of 500 nl/min. Peptides were separated with a gradient of 5–40% solvent B over 90 min, followed by a gradient of 40–60% for 10 min and 60–80% over 5 min at a flow rate of 250 nl/min in the EASY-nLC 1000 system. The Q Exactive was operated in the data-dependent acquisition (DDA) mode to automatically switch between full scan MS and MS/MS acquisition. Survey full scan MS spectra (from m/z 300-1150) were analyzed in the Orbitrap detector with resolution R = 35,000 at m/z 400. The 15 most intense peptide ions with charge states 2+ were sequentially isolated to a target value of 3 × 10^6^ and fragmented by Higher Energy Collision Dissociation (HCD), with a normalized collision energy setting of 25%. The maximum allowed ion accumulation times were 20 ms for full scans and 50 ms for MS/MS and the target value for MS/MS was set to 10^6^. The dynamic exclusion time was set to 20 s.

### MS Raw Data Processing for Protein Identification and Quantification

MS raw data were analyzed with the integrated MaxQuant software v1.6.2.10, using the Andromeda search engine (Cox et al., 2011; Tyanova et al., 2016). The 2020_06 version of the UniProt Human sequence database (UP000005640) was used for peptide identification. In MaxQuant, the estimated FDR of all peptide identifications was set to a maximum of 1%. The main search was performed with a mass tolerance of 4.5 parts per million (ppm). Enzyme specificity was set to Trypsin/P. A maximum of three missed cleavages was permitted, and the minimum peptide length was fixed at seven amino acids. Carbamidomethylation of cysteine was set as a fixed modification. To assign and quantify SILAC methyl-peptides, all MS raw data were processed indicating N-terminal acetylation, Methionine oxidation, mono-methyl-K/R, and di-methyl-K/R as variable modifications. The MaxQuant proteinGroups.txt output file was then filtered: potential contaminants and reverse sequences were removed, and proteins were required to be identified by at least 2 peptides, one of which unique, and to have an Andromeda score ≥25. Last, proteins SILAC H/L and M/L ratios in the interface were normalized on the respective protein SILAC H/L and M/L ratios in WCE, both extracted from the proteinGroups.txt MaxQuant output file. This normalization allowed to discriminate between changes of protein level within the interface fraction (as the hypothetical consequence of a different interaction with cognate RNAs) and the mere protein expression changes following transcriptional changes induced by pharmacological inhibition of PRMTs.

### Functional Analysis for Characterization of the Interface

Gene Ontology enrichment analysis was performed with GOrilla and Revigo (Eden et al., 2009; Supek et al., 2011). Analysis of protein-protein interaction network and analysis of protein domains were carried out through the STRING plugin of Cytoscape (Shannon et al., 2003; Szklarczyk et al., 2019).

### Motif Analysis

Motif analysis was performed using the pLogo web application (O'Shea et al., 2013). For each R in the human proteome, a 5-amino acid sequence window centered on that R was extracted from the 2020_06 version of the SwissProt human database. Sequence windows from proteins in the interface and in the WCE were then provided to pLogo as foreground sequences, while sequence windows from the remaining proteins were used as background.

### Statistical Analysis of the Stable Isotope Labelling with Amino acids in Cell Culture -Based Quantitative Proteomics Data

To define up- or down-regulated proteins by MS023 or GSK591, we used mean (μ) and SD (σ) based on the distribution of the proteins SILAC ratios calculated separately in the forward and reverse experiments for the DMSO condition and applied a μ±1σ cutoff to the protein ratio distributions in each replicate. To determine whether the abundance of the interface proteins was significantly affected by PRMT inhibitors compared to their expression level in the corresponding whole cell extracts, Fisher’s exact tests were performed with Python SciPy package. Clusters of regulated proteins were defined with Ward’s method (Ward, 1963).

### Western Blot Analysis

For Western Blot analysis, protein extracts were lysed in urea lysis buffer (9 M urea, 20 mM Hepes (pH 8.0)), supplemented with 1× cocktail of proteases and phosphatase inhibitors (Roche) from HeLa cells and quantified by BCA assay (Pierce BCA Protein assay kit). Equal protein amounts were separated by SDS-PAGE electrophoresis and transferred on Transfer membrane (Immobilon-P, Merck Millipore) by wet-transfer method. Membrane blocking with 10% BSA/TBS 0.1% Tween-20 for 1 h at RT was followed by overnight incubation with the primary antibodies and subsequently with the HRP-conjugated secondary antibodies, for 1 h (Cell Signaling Technology). Proteins were detected by ECL (Bio-Rad). The following primary antibodies were used: anti-vinculin (V9131, 1:5000) was purchased from Merk Life Science; anti-RPS2 (A303-794A, 1:5000), anti-CCT5 (A300-421A 1:5000), anti-LDHB (A304-7070A 1:5000) and anti-PRMT4 (A300-421A 1:5000) were purchase from Bethyl Laboratories; anti-NONO (SC-376865, 1:500), anti-hnRNPH3 (SC-376416, 1:500) and anti-alpha-tubulin (SC-32293, 1:1000) were purchase from Santa Cruz Biotechnology; anti-HSP90AA1 (AB2928, 1:1000), anti-HuR (AB136542, 1:1000), anti-HMGB1 (AB79823, 1:5000), anti-TIA1 (AB140595, 1:2000), anti-H4R3me2s (AB5923, 1:1000), anti-H3R2me2a (AB9147061:1000), anti-H3R17me2a (AB8284 1:1000), anti-PRMT6 (AB 47244 1:1000) and anti-total histone 4 (AB7311 1:2000) were purchase from Abcam; anti-H4R3me2a (61988, 1:500) was purchase from Active Motifs; anti-ADMA (ASYM24 07-414, 1:1000) and anti-SDMA (SYM10 07-412, 1:2000) were purchase from Millipore; anti-MMA (D5A12; 1:1000) was purchase from Cell Signaling Technology; anti-pan -14-3-3 (MA5-1224, 1:2000) was purchase from Thermo Fisher Scientific.

### Protein Arginine Methyltransferase 1 Knock-Down by shRNA

PRMT1 knock-down (KD) in Hela cells was obtained using a second-generation pLKO lentiviral vectors, in which two distinct shRNAs targeting PRMT1 were cloned:

5′-CCG​GCA​GTA​CAA​AGA​CTA​CAA-3′ (sh#1, PRMT1), 5′-GTG​TTC​CAG​TAT​CTC​TGA​TTA-3′ (sh#2, PRMT1).

The pLKO scamble shRNA was used as negative control. To obtain PRMT1 depletion, HeLa cells were transduced using lentiviruses whose stocks were produced by transient CaCl_2_ transfection of HEK293 cells with the packaging plasmid pCMV-DR8.74, the envelope plasmid pMD2G-VSVG and the respective transfer gene-carrying vector. After 48 h from transfection, the supernatant containing the virus was ultra-centrifuged and added to the HeLa medium. Transduced Hela cell were then selected by incubation with 1 µg/ml puromycine for 48 h and subsequent used for the downstream applications.

### RNA Interactome Capture (RIC) Experiment Followed by Mass Spectrometry Analysis or Western Blot Profiling

The RNA pull-down was performed as described in (Castello et al., 2013). Briefly, SILAC-labelled HeLa cells were treated with DMSO or MS023 10 µM or GSK591 5 µM for 48h and then were harvested and UV-crosslinked at 254 nm at a dose of 40 J/m2 using a Stratalinker 2400 UV cross-linker (Stratagene, La Jolla, CA). A small aliquot (corresponding to about 10%) of sample from each treatment was saved for WB analysis while the remaining was mixed in 1:1:1 proportion with samples from the other conditions. Cell were then lysed and poly(A)-mRNAs were pulled down 3 times (each time using the flow-through from the previous pull-down) using Dynabeads® Oligo (dT)_25_ (Thermo Fisher Scientific). Poly(A)-mRNA-associated proteins were eluted from the beads following the manufacturer’s instruction and subsequently processed by in-solution digestion prior to LC-MS/MS.

WB profiling of candidate proteins from OOPS and RIC were also carried out for validation of the quantitative MS-proteomics data. For the WB analysis each band intensity in each condition was measured with FiJi software (http://www.yorku.ca/yisheng/Internal/Protocols/ImageJ.pdf) and subsequently normalized at four different leves:

1. In the WCE, each band was normalized on the vinculin as loading control;
2. In the interface or RIC fractions, each band was normalized on HuR or NONO, selected as loading controls for the interface/RIC, because they resulted unchanging from quantitative proteomics analyses;
3. For each treatment, the intensity in the interface/RIC was normalized over the corresponding in the WCE, to discern the different abundance within these fractions from mere protein expression changes;
4. After the previous three normalizations, the intensity for each treatment was normalized over DMSO.

### Protein Immunoprecipitation

Protein immunoprecipitation (IP) was performed starting from 500 µg of HeLa cell extract, 5% of cell extract was loaded as input. Briefly, 30 × 10^6^ HeLa cells were harvested, washed twice with cold PBS and re-suspended in 2 volumes of RIPA Buffer (10 mM Tris pH 8, 150 nM NaCl, 0.1% SDS, 1% Triton X-100, 1 mM EDTA, 0.1% Na-Deoxycholate,1 mM PMSF,1 mM DTT and 1x Proteases and Phosphatase Inhibitors cocktail (Roche), supplemented with 10K U of Benzonase (Merck Life Science). The suspension was rotated on wheel for 45 min at RT (vortexing every-10 min), centrifuged at 12.000 g for 1 h at 4°C and the supernatant was transferred into a new Eppendorf tube; proteins were quantified by BCA colorimetric assay (Pierce BCA Protein assay kit). The protein lysate was rotated at 4°C overnight with 8 µg of anti-pan -14-3-3 (MA5-1224 Thermo Fisher Scientific) or 5 μg of anti-LDHB (A304-770A Bethyl Laboratories) each for 500 µg of protein extract. In parallel, G-protein-coupled magnetic beads (Dynabeads, Thermo Fisher Scientific) were blocked with a blocking solution (0.5% BSA in TBS supplemented with 1% Triton X-100) and rotated at 4°C overnight. The following day, the beads were added to the lysate in 1:100 proportion with a primary antibodies and incubated for 3 h at 4°C on a wheel; the captured immuno-complexes were washed 4 times with the RIPA Buffer and then incubated for 10 min at 95° with LSD sample Buffer supplemented with 100 mM DTT in order to elute the immunoprecipitated proteins for subsequent analyses.

### Confocal Immunofluorescence Experiments

Cells were plated on glass coverslips for 24 h and grown with DMSO or 10 µM MS023 or 5 µM GSK591 for 48 h or 400 µM NaAsO_2_ for 30 min and 0.8 mM modified uridine (EU) for 48 h. For MS023 and NaAsO_2_ tretaments, each experiment was carried out in duplicate and one of the two experiments was subsequently treated with 5% 1,6-Hexanediol for 10 min. Then, cells were washed with 1x PSB, fixed in 4% paraformaldehyde for 20 min at RT, permeabilized with 0.1% Triton X-100 in PBS for 5 min on ice and the “click” reaction was carried out to conjugate the incorporated EU with Alexa 594 Fluor according to the manufacture instruction (Click-iT™ RNA Alexa Fluor™ 594 Imaging Kit, Thermo Fisher Scientific). Then, cells were incubated with 2% BSA in PBS for 30 min at RT and subsequently with the primary antibody in PBS containing 2% BSA overnight at 4°C. After being washed, the primary antibodies were removed and cells were incubated with the antibody anti-G3BP1 (BD 611126 1:400) for 3 h at RT. After three additional washes, cells were stained with Rabbit Alexa Fluor 488 secondary antibody (for the protein of interest) or with Mouse Alexa Fluor 647 secondary antibody (for G3BP1) (Molecular Probes, Eugene, OR, United States), both diluted 1:400 in PBS containing 2% BSA for 1 h at RT. Nuclei were stained with DAPI (Invitrogen). Images were acquired with a Leica TCS SP8 confocal microscope (Leica Microsystems, Heerbrugg, Switzerland).

### Confocal Imaging and Analysis

To evaluate the percentage of cells with stress granules, samples were acquired with a Nikon CSU-W1 spinning disk using a 60X/1.4NA objective lens, a 50 um-pinhole disk, solid state lasers, a multiband dichroic mirror and a fast-rotating emission filters wheel. Eighty-one fields of view (FOV) were automatically acquired for each sample with an autofocus routine on the DAPI channel. A Z-stack of 7 optical sections with a step size of 0.6 μm together with the emissions from the 4 fluorophores (DAPI, AlexaFluor488, AlexaFluor594 and AlexaFluor647) were acquired in each FOV with a pixel size of 108 × 108 nm (2048 × 2048 pixels per FOV). The acquired images were analysed with a custom-made FiJi/ImageJ (Schindelin et al., 2012) macro. Briefly, the DAPI channel was used to identify the relevant area of each cell (cell area) using the Voronoi filter on the maximum intensity projections. In each cell area the presence of a nucleus was evaluated, and the cell areas without any nucleus or with more than one, were discarded. Then, a band of 12 microns was created around each nucleus (cytoplasmic area), the G3BP1 signal was used to segment the stress granules using a fixed threshold in all samples and the objects inside the cytoplasmic area (stress granules) were counted in each cell. For each sample, the number of cells with at least one stress granule was considered. To evaluate the colocalization between G3BP1 and the RBP signals, single optical sections per sample were acquired with a Leica SP8 laser scanning confocal microscope equipped with a 405 nm and 561 solid state lasers, an Argon and a HeNe lasers, Hybrid detectors and a motorized stage. More than 15 FOV per sample were acquired using a 63X/1.4NA objective lens with a pixel size of 45 nm (2048 × 2048 pixels per FOV). The co-localization indices were calculated in a 10um-thick band around each nucleus (cytoplasmic area) thanks to a custom-made FiJi/ImageJ macro and the JaCoP plug-in (Bolte and Cordelieres, 2006). In all experimental conditions, the M1 coefficient (the fraction of RBP signal colocalizing with G3BP1 signal) was used as indication of colocalization between RBP and stress granules. The Huang and the Max Entropy algorithms were used to automatically find the thresholds for RBP and G3BP1 signals, respectively.

## Data Availability

The datasets presented in this study can be found in online repositories. The mass spectrometry proteomics data have been deposited to the ProteomeXchange Consortium via the PRIDE (Perez-Riverol et al., 2019) partner repository with the dataset identifier PXD024601.
