# Supplementary figures and images for: Systematic Analysis of the Impact of R-Methylation on RBPs-RNA Interactions: A Proteomic Approach
Source: Front Mol Biosci. 2021 Sep 7;8:688973. doi: 10.3389/fmolb.2021.688973 (PMC8454774; doi:10.3389/fmolb.2021.688973)

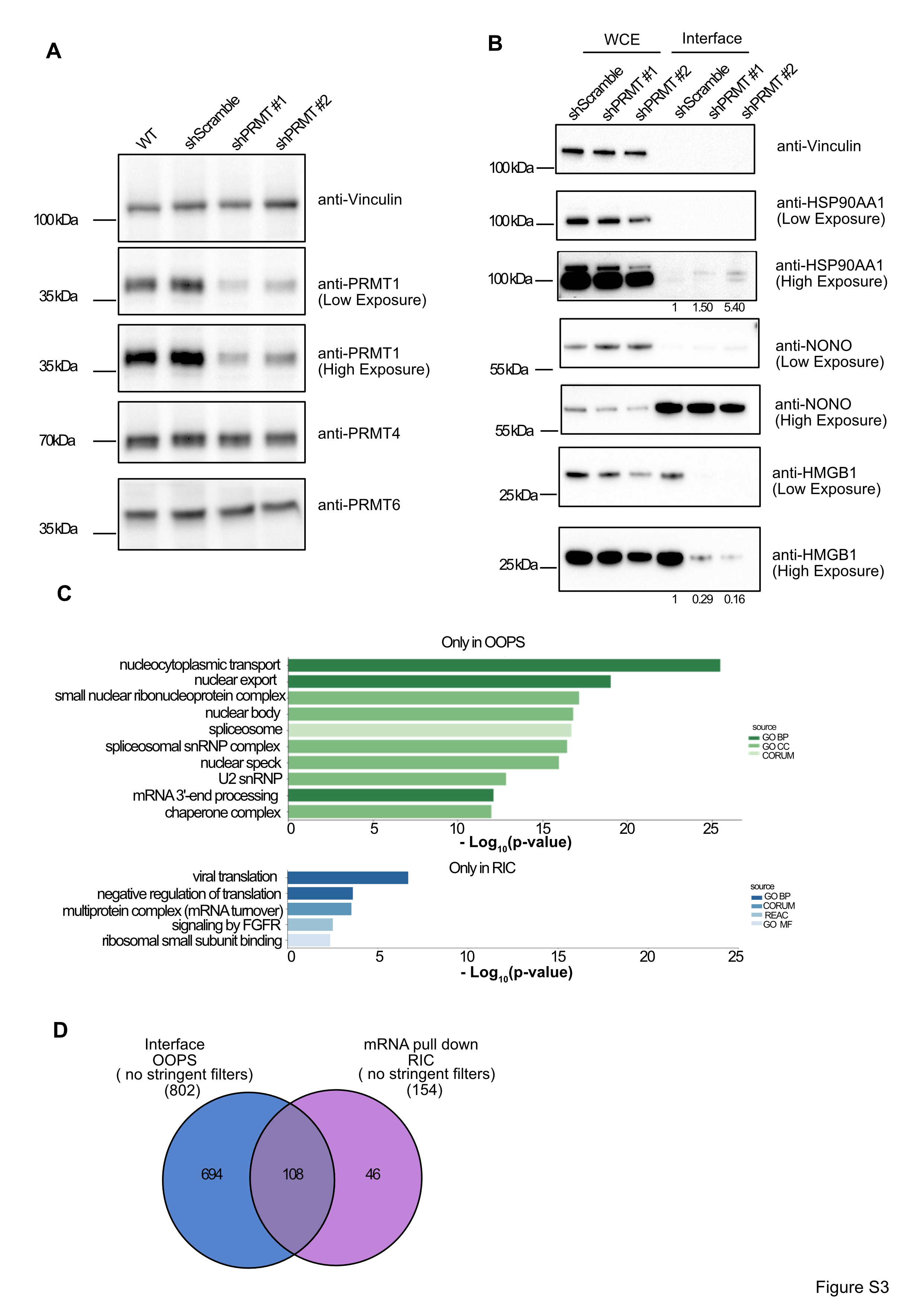

Supplement: Supplementary file 1 [file Image3.TIFF]

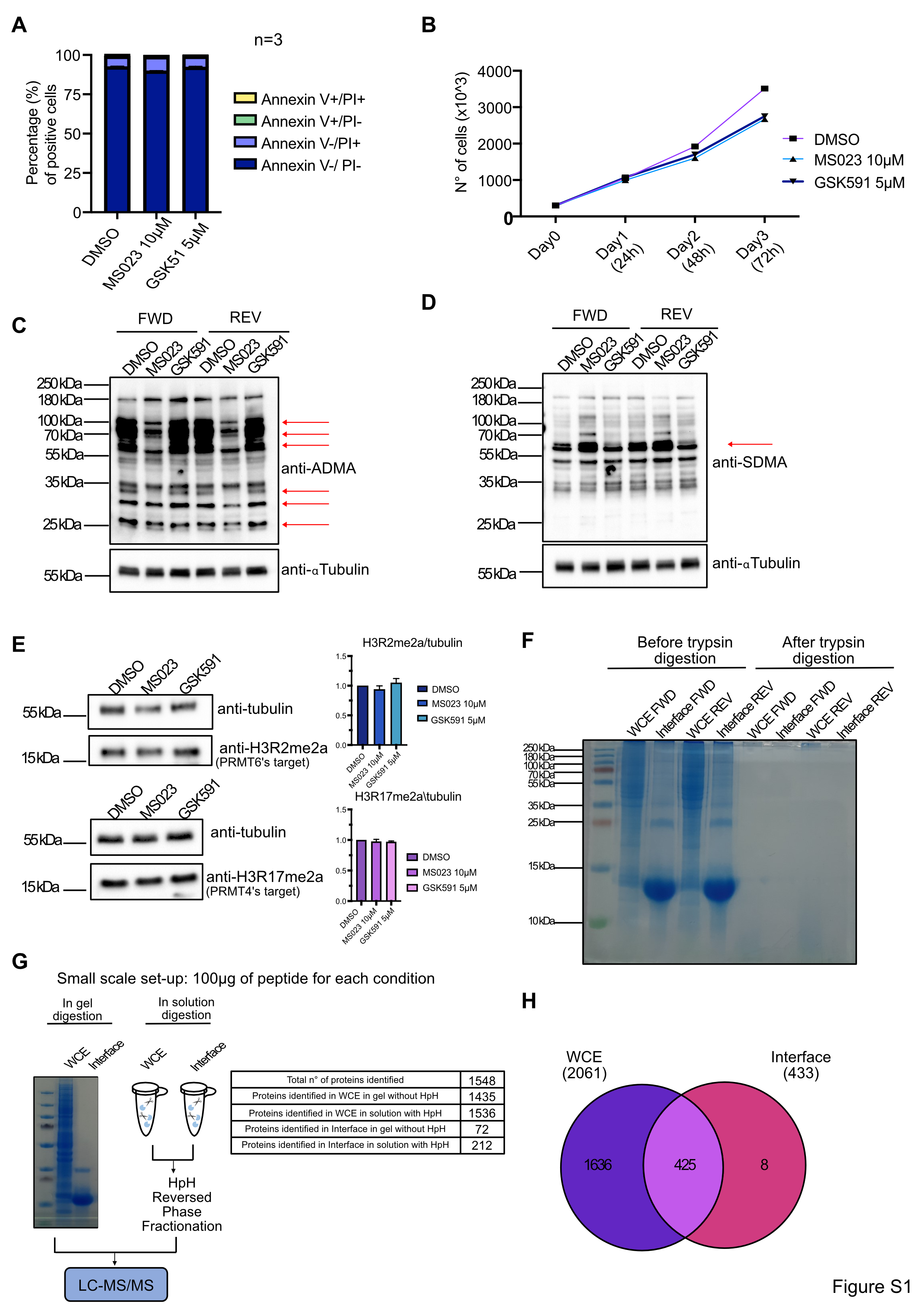

Supplement: Supplementary file 3 [file Image1.TIFF]

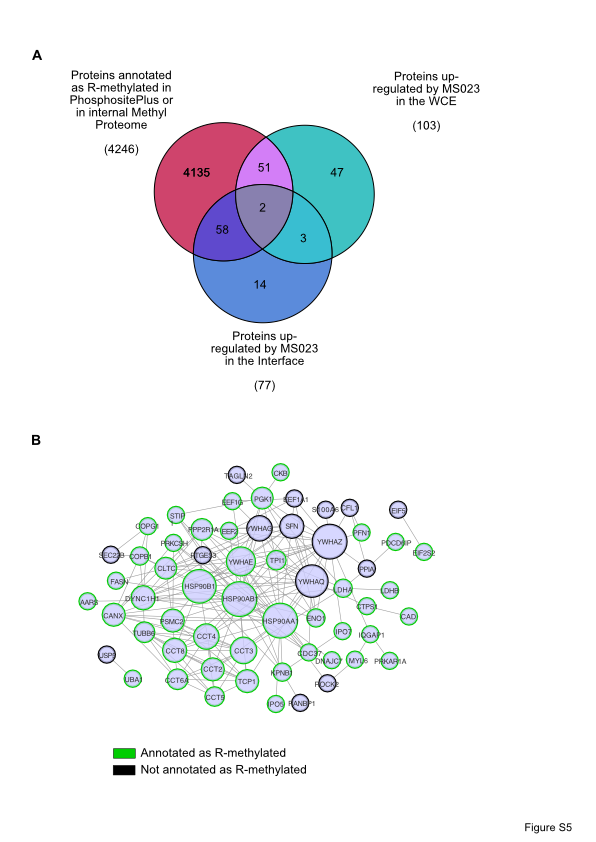

Supplement: Supplementary file 4 [file Image5.TIFF]

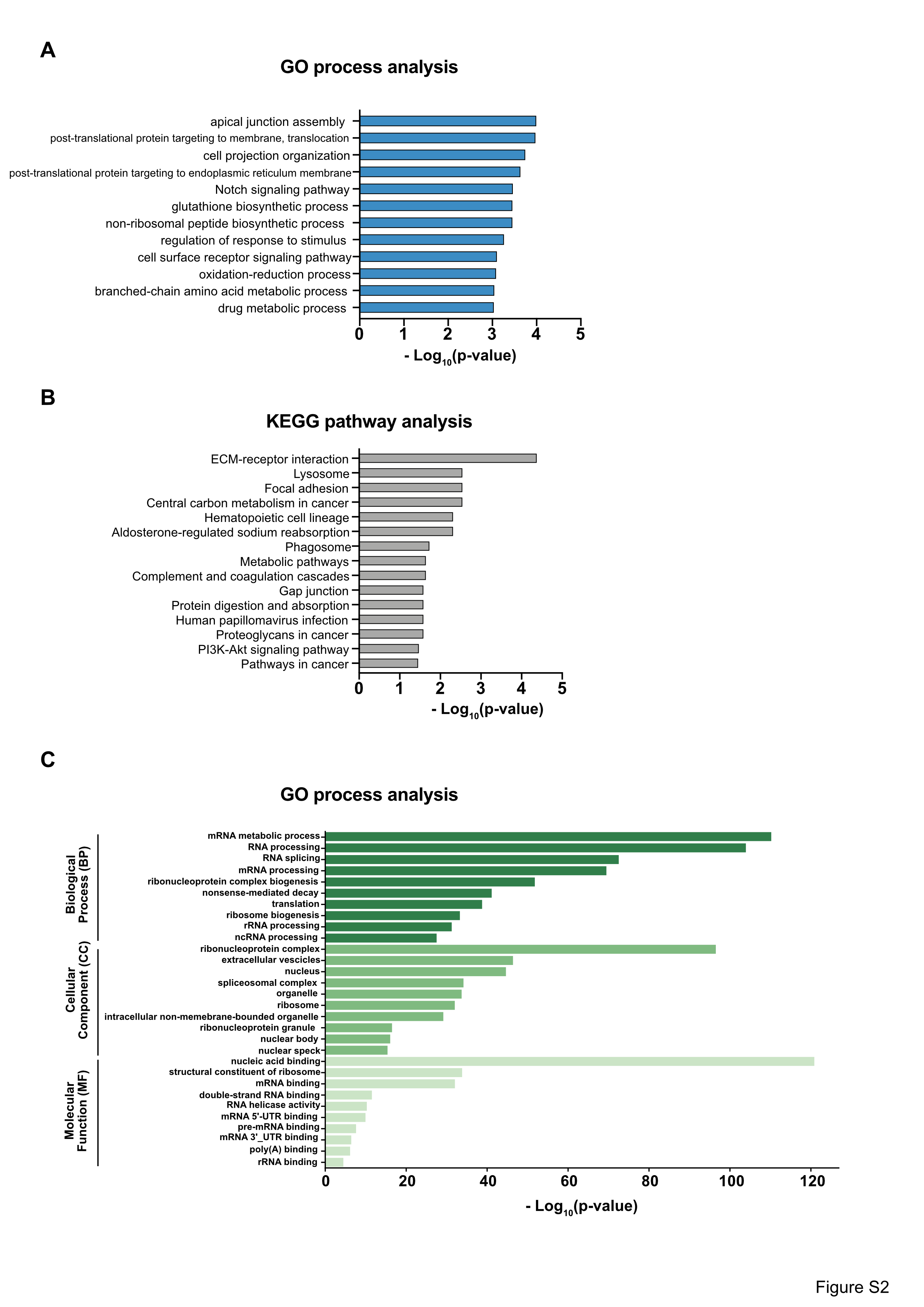

Supplement: Supplementary file 6 [file Image2.TIFF]

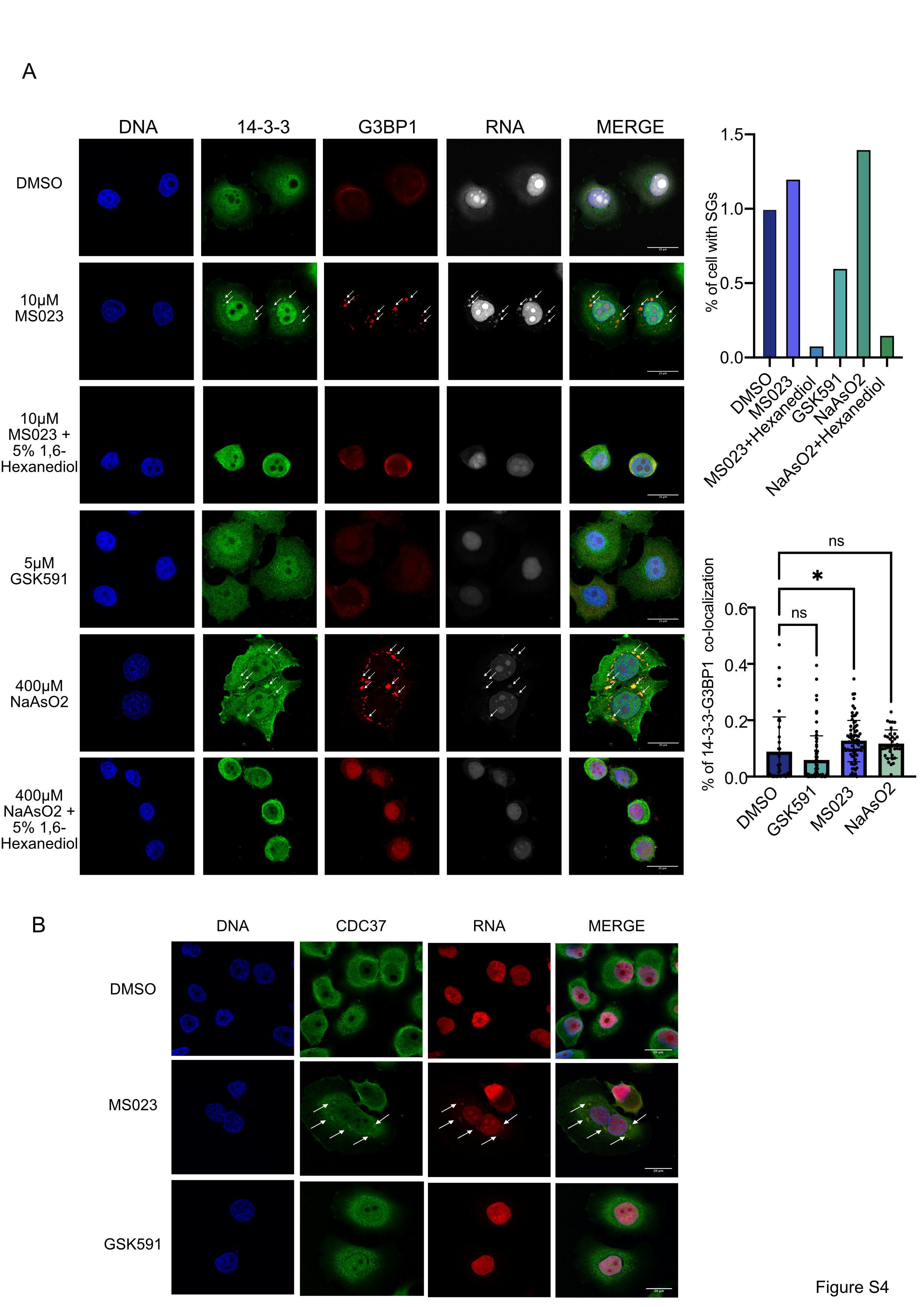

Supplement: Supplementary file 7 [file Image4.TIFF]
