# Supplementary material for: Systematic Analysis of the Impact of R-Methylation on RBPs-RNA Interactions: A Proteomic Approach
Source: Front Mol Biosci. 2021 Sep 7;8:688973. doi: 10.3389/fmolb.2021.688973 (PMC8454774; doi:10.3389/fmolb.2021.688973)
Supplement: Supplementary file 8 [file DataSheet1.docx]

Supplementary Material

# Supplementary Data

This Supplementary File includes:

- Supplementary Figure 1 (Figure S1), Supplementary Figure 2 (Figure S2), Supplementary Figure 3 (Figure S3), Supplementary Figure 4 (Figure S4) and Supplementary Figure 5 (Figure S5)
- Supplementary Table 1 (Table S1) and Supplementary Table 2 (Table S2)
- Supplementary Material and Methods

# Supplementary Figures and Tables

## Supplementary Figures

## Supplementary Figure 1. Experimental controls of the proteomic workflow

**A.** Cell death assay, performed by flow cytometry analysis of Annexin V and Propidium Iodide (PI) expression upon DMSO, 10µM MS023 and 5µM GSK591 treatment for 48h, in triplicate. Annexin V+ cells represent apoptotic cells, PI+ cells represent necrotic cells. **B.** Counts of living cells by trypan blue staining at each time point (0h, 24h, 48h, 72h) upon DMSO, 10µM MS023 and 5µM GSK591 treatment. **C.** WB analysis of global protein ADMA state by probing whole protein extract from HeLa cells treated with DMSO, 10µM MS023 and 5µM GSK591, both in the FWD and in the REV SILAC experiment, using the pan antibody anti-ADMA and anti-α- tubulin as loading control. Red arrows indicate specific changes in ADMA levels **D.** WB analysis of global protein SDMA probing whole extract from HeLa cells treated with DMSO, 10µM MS023 and 5µM GSK591, both in FWD and in REV experiment, with the pan antibody anti-SDMA; α-tubulin was then re-probed on the same filter as loading control. Red arrows indicate specific changes in SDMA levels **E.** WB profiling of post translational modification occurring on histones to monitor the activity of other type I PRMT enzyme upon MS023 treatment: histone 4 arginine 2 asymmetrically di-methylated (H3R2me2a) and histone 3 arginine 17 asymmetrically di-methylated (H3R17me2a) were probed as target of the activity of PRMT6 and PRMT4, respectively. **F.** SDS-PAGE gel stained with Blue Coomassie to detect proteins before and after in-solution trypsin digestion, from WCE and interface fraction, both for the FWD and REW experiment. **G.** Schematic workflow and summary table of identified proteins comparing in-gel digestion without HpH RP chromatographic fractionation and in-solution digestion coupled with HpH RP chromatography **H.** Comparison of the total number of proteins identified in the WCE and in the interface fraction enriched by OOPS.

**Supplementary Figure 2. Gene ontology (GO) analysis of proteins enriched in the interface fraction and not annotated in other databases for RBPs**

**A.** Bar-graph representation of the most enriched biological process terms from the GO analysis of the 63 proteins identified in the interface fraction not overlapping with the EuRBPDB database ([Liao et al., 2020](#_ENREF_2)) and the list of RBPs published in ([Queiroz et al., 2019](#_ENREF_3)), as illustrated in Figure 2. Functional analysis was performed by Gene Ontology enRIchment anaLysis and visuaLizAtion (GOrilla) tool. **B.** Bar-graph representation of the pathways over-represented in the same set of proteins, using KEGG pathway database. **C.** Bar-graph representation of the top 10 most enriched terms for each of the 3 GO categories in the interface fraction: Biological Process (BP), Cellular Component (CC) and Molecular Function (MF). This figure is related to main Fig. 2B.

**Supplementary Figure 3. Validation of the PRMT1 mediated RBP-RNA binding dynamicity**

**A.** WB profiling of PRMT1, PRMT4 and PRMT6 upon PRMT1 knock-down by two distinct shRNA constructs. **B.** WB profiling of HSP90AA1 and HMG1 protein in WCE and interface fraction following OOPS in PRMT1 KD and control (scramble shRNA) cells. Quantification of the band intensity in the interface for HSP90AA1 and HMHB1 is displayed, both normalized as described in the material and methods of the main text. **C.** Comparative GO analysis of the most enriched biological process terms from proteins identified in either OOPS only or RIC only. **D.** Venn-diagram displaying the intersection of proteins identified in OOPS and RIC when less stringent filtering criteria were applied, as described in the main text. This figure is related to main Fig. 4C and D.

**Supplementary Figure 4. Immunofluorescence analysis of selected RBPs up-regulated by MS023 in the OOPS-MS experiment**

**A.** Representative images of IF analysis of 14-3-3 proteins in HeLa cells treated with the following compounds: DMSO, 10 μM MS023 and 5 μM GSK591 for 48 hours; 10 μM MS023 for 48 hours, followed by 10 min treatment with 5% 1,6-Hexanediol; 400μM NaAsO2 for 30 min; 400μM NaAsO2 for 30 min followed by 10 min treatment with 5% 1,6-Hexanediol. Immunostaining of RNA was performed with the Click-iT™ RNA Alexa Fluor™ 594 Imaging Kit. DAPI staining was used for DNA visualization. G3BP1 staining was used as positive control for SGs formation. DAPI, LDHB, G3BP1 and RNA staining and the respective merged images are displayed. Images were taken by SP8OBS confocal microscopy using a 60× oil objective, and a scale bar of 25 μM are included in the merged figure. White arrows indicate co-localization of target RBP, G3BP1 and RNA. The first bar-graph represents the percentage of cells with more than 1 G3BP1+ granule and all the treatments were normalized on the DMSO. The second bar-graph describes the percentage of co-localization between G3BP1 and 14-3-3 in the SGs in each condition. All the treatments were normalized over the DMSO. Statistical significance was calculated by Student’s t-test (*p < 0.05). **B.** Representative IF images of CDC37 protein in Hela cells treated with the following compounds: DMSO, 10 μM MS023 and 5 μM GSK591 for 48 hours. Immunostaining of RNA was performed with the Click-iT™ RNA Alexa Fluor™ 594 Imaging Kit. DAPI staining was used for nuclei visualization. DAPI, CDC37 and RNA staining and the respective merged images are displayed. Images were taken by SP8 confocal microscopy using a 60× oil objective, and a scale bar of 20 μM are included in the merged figure. White arrows indicate co-localization of target RBP and RNA

**Supplementary Figure 5. Protein-protein interaction network of the proteins MS023-regulated**

**A.** Venn-diagram illustrating the intersection of the proteins up-regulated by MS023 in the WCE, in the interface fraction and annotated as R-methylated in PhospshositePlus Database ([Hornbeck et al., 2015](#_ENREF_1)) and in our internal high-confidence methyl-proteome dataset (Massignani et al, *in preparation*). **B.** Protein-protein interaction network of proteins modulated in the interface fraction by MS023 treatment. The size of each node is proportional to the number of interacting proteins. Proteins annotated in PhospshositePlus database or in our internal high-confidence methyl-proteome are circled in green; proteins not annotated in the aforementioned datasets are circled in black.

## Supplementary Tables

**Table S1**. **Proteins regulated upon MS023 and GSK591 treatment in OOPS experiment**

MS-based protein profiling in response to MS023 and GSK591 in both WCE and interface fraction from OOPS, expressed as Log_2_ SILAC protein ratios, both in the FWD and REV experiments. **Datasheet #1** (Experimental design): scheme of the SILAC-OOPS experimental design. **Datasheet #2** (All Raw Data) reports all proteins identified with at least 2 peptides, one of which unique, and Andromeda score ≥25. **Datasheet #3** (Significantly regulated WCE) contains the proteins significantly regulated (±1σ for each SILAC protein distribution) in the WCE. **Datasheet #4** (Significantly regulated OOPS) contains the proteins significantly regulated (±1σ for each SILAC protein distribution) in the interface fraction. **Datasheet #5** (OOPS normalized over WCE) shows the Log_2_ SILAC protein ratios in FWD and REV replicates of each SILAC experiment, normalized on the corresponding protein ratios in the WCE. **#Datasheet 6** (OOPS relaxed) contains all proteins identified in the interface fraction, when the stringent filters (identification by at least 2 peptides, one of which unique and Andromeda score ≥25) were relaxed. Reverse identifications and contaminants have been removed.

**Table S2**. **Regulated proteins upon MS023 and GSK591 treatment in the RIC experiment**

MS-based protein profiling in response to MS023 and GSK591 in both WCE and RNA pull-down sample from RIC, expressed as Log_2_ SILAC protein ratios, both for the FWD and REV experiment.

**Datasheet #1** (Experimental design): scheme of the SILAC-RIC experimental design. **Datasheet #2** (All Raw Data) reports all proteins identified with at least 2 peptides, one of which unique, and andromeda score ≥25. **Datasheet #3** (RIC no stringent normalized on WCE) shows the Log_2_ SILAC ratios in the forward and reverse replicate of each SILAC experiment normalized on the corresponding channel in the WCE. **Datasheet #4** (Comparison RIC vs OOPS) contains all proteins identified both in the RIC and in the OOPS experiments in at least one of the two replicates by comparing the two datasets defined by less stringent criteria.

# Supplementary Material and Methods related to Supplementary results

**Cell death assay**. HeLa cells were grown for 48 hours in the presence of DMSO, 10µM MS023, 5µM GSK591; upon harvesting, apoptosis was measured by evaluation of FITC-conjugated Annexin V (Thermo Fisher Scientific) and propidium iodide (Thermo Fisher Scientific), following the manufacturers’ protocols. Cells were measured using the FACSCalibur platform (BD Bioscience) and analyzed using the FlowJo software. Statistical analysis was performed using ANOVA (Microsoft Excel; Microsoft, Redmond, WA, USA). Biological replicates n = 3.

**Bioinformatic tools and GO analysis**. Gene Ontology (GO) analysis for Biological Process and pathway enrichment were performed by Gene Ontology enRIchment anaLysis and visuaLizAtion (GOrilla) tool (http://cbl-gorilla.cs.technion.ac.il/) and KEGG pathway database (https://www.genome.jp/kegg/pathway.html), respectively. Protein-protein interactions network was generated with Cytoscape open source software platform for visualizing molecular interaction networks and biological pathways (https://cytoscape.org/index.html).

# Supplementary References

# HORNBECK, P. V., ZHANG, B., MURRAY, B., KORNHAUSER, J. M., LATHAM, V. & SKRZYPEK, E. 2015. PhosphoSitePlus, 2014: mutations, PTMs and recalibrations. *Nucleic Acids Res,* 43, D512-20.

# LIAO, J. Y., YANG, B., ZHANG, Y. C., WANG, X. J., YE, Y., PENG, J. W., YANG, Z. Z., HE, J. H., ZHANG, Y., HU, K., LIN, D. C. & YIN, D. 2020. EuRBPDB: a comprehensive resource for annotation, functional and oncological investigation of eukaryotic RNA binding proteins (RBPs). *Nucleic Acids Res,* 48, D307-D313.

# QUEIROZ, R. M. L., SMITH, T., VILLANUEVA, E., MARTI-SOLANO, M., MONTI, M., PIZZINGA, M., MIREA, D. M., RAMAKRISHNA, M., HARVEY, R. F., DEZI, V., THOMAS, G. H., WILLIS, A. E. & LILLEY, K. S. 2019. Comprehensive identification of RNA-protein interactions in any organism using orthogonal organic phase separation (OOPS). *Nat Biotechnol,* 37, 169-178.
